# Characterization of pathogenic monoclonal autoantibodies derived from muscle-specific kinase myasthenia gravis patients

**DOI:** 10.1172/jci.insight.127167

**Published:** 2019-06-20

**Authors:** Kazushiro Takata, Panos Stathopoulos, Michelangelo Cao, Marina Mané-Damas, Miriam L. Fichtner, Erik S. Benotti, Leslie Jacobson, Patrick Waters, Sarosh R. Irani, Pilar Martinez-Martinez, David Beeson, Mario Losen, Angela Vincent, Richard J. Nowak, Kevin C. O’Connor

**Affiliations:** 1 Department of Neurology and; 2 Department of Immunobiology, Yale School of Medicine, Yale University, New Haven, Connecticut, USA.; 3 Neurosciences Group, Weatherall Institute of Molecular Medicine and Nuffield Department of Clinical Neurosciences, Oxford, England.; 4 Department of Psychiatry and Neuropsychology, School for Mental Health and Neuroscience, Maastricht University, Maastricht, Netherlands.; 5 Oxford Autoimmune Neurology Group, Nuffield Department of Clinical Neurosciences, John Radcliffe Hospital, University of Oxford, Oxford, England.

**Keywords:** Autoimmunity, Immunology, Autoimmune diseases, B cells, Neuromuscular disease

## Abstract

Myasthenia gravis (MG) is a chronic autoimmune disorder characterized by muscle weakness and caused by pathogenic autoantibodies that bind to membrane proteins at the neuromuscular junction. Most patients have autoantibodies against the acetylcholine receptor (AChR), but a subset of patients have autoantibodies against muscle-specific tyrosine kinase (MuSK) instead. MuSK is an essential component of the pathway responsible for synaptic differentiation, which is activated by nerve-released agrin. Through binding MuSK, serum-derived autoantibodies inhibit agrin-induced MuSK autophosphorylation, impair clustering of AChRs, and block neuromuscular transmission. We sought to establish individual MuSK autoantibody clones so that the autoimmune mechanisms could be better understood. We isolated MuSK autoantibody-expressing B cells from 6 MuSK MG patients using a fluorescently tagged MuSK antigen multimer, then generated a panel of human monoclonal autoantibodies (mAbs) from these cells. Here we focused on 3 highly specific mAbs that bound quantitatively to MuSK in solution, to MuSK-expressing HEK cells, and at mouse neuromuscular junctions, where they colocalized with AChRs. These 3 IgG isotype mAbs (2 IgG4 and 1 IgG3 subclass) recognized the Ig-like domain 2 of MuSK. The mAbs inhibited AChR clustering, but intriguingly, they enhanced rather than inhibited MuSK phosphorylation, which suggests an alternative mechanism for inhibiting AChR clustering.

## Abstract 2

A fluorescent tetrameric antigen allows isolation of human myasthenia gravis monoclonal antibodies that interrupt acetylcholine receptor signaling.

## Introduction

Patients with myasthenia gravis (MG) experience skeletal muscle weakness, worsened by activity. Typically, they present with ocular muscle weakness, which then generalizes to involve limb muscles and bulbar and respiratory muscles in particular (1, 2). The molecular immunopathology of MG is directly attributed to the presence of circulating autoantibodies specifically targeting extracellular domains of postsynaptic membrane proteins at the neuromuscular junction (NMJ; refs. 1, 3). The disease has multiple subtypes, defined by different autoantibody targets (4–7). Autoantibodies against the acetylcholine receptor (AChR), which are present in around 85% of patients, are mainly IgG1 and cause loss of AChRs via divalent binding, which leads to internalization of AChRs and complement-mediated damage to the NMJ (1, 8). Some of the 15% of patients without AChR autoantibodies have, instead, autoantibodies against muscle-specific tyrosine kinase (MuSK) (7) or, less commonly, have low-density lipoprotein receptor–related protein 4 (LRP4; refs. 9, 10). The MuSK autoantibody form of MG can be severe because it usually involves mainly bulbar muscles (11), which affects speaking, chewing, swallowing, and breathing, and can cause permanent muscle atrophy over time (12, 13). MuSK autoantibodies are particularly interesting because they are predominantly (14) of the non–complement-activating IgG4 subclass; the subclass can be functionally monovalent for antigen binding and hence does not cross-link its antigen (15). Yet MuSK autoantibodies are demonstrably pathogenic, which can be established by passive transfer of the human disease phenotype to mice by injection of patients’ IgG or active immunization with MuSK (16–18).

MuSK is an essential component of the agrin/LRP4/MuSK/downstream of tyrosine kinase 7 (DOK7) pathway that is responsible for clustering of AChRs at the NMJ, both during development and in mature muscle (19). Serum-derived MuSK autoantibodies mainly recognize the N-terminal Ig-like domain 1 of MuSK and prevent the binding of LRP4 to MuSK (20–22). As a result, autophosphorylation of MuSK is inhibited and DOK7 is not recruited to complete the pathway. These effects can be demonstrated in the mouse myotube-forming C2C12 cell line, where MuSK autoantibodies prevent agrin/LRP4–induced clustering of AChRs. In this model, isolated antigen-binding fragments (Fabs) from MuSK-specific antibodies are sufficient to inhibit AChR clustering (23). In contrast, AChR autoantibodies require divalent binding to cause loss of AChRs (8, 24, 25). Although some of the mechanisms underlying MuSK autoantibody–associated MG appear well understood, patients’ autoantibodies are heterogeneous. For instance, IgG1, IgG2, and IgG3 MuSK autoantibodies exist in most patients, and their pathogenic mechanisms have not been well studied. Moreover, it is unclear whether autoantibodies against domains other than the first Ig-like domain in MuSK may contribute to disease. We sought to establish individual MuSK IgG clones so that the mechanisms in this disease could be better analyzed both in vitro and in vivo.

All forms of MG improve with immunotherapies, but B cell depletion with a therapeutic monoclonal autoantibody (mAb; rituximab) against the B cell marker CD20 leads to substantial reductions in MuSK autoantibodies and relatively quick clinical improvement (11, 26, 27). The success of anti-CD20 therapy suggests that the autoantibodies are derived from circulating MuSK-specific B cells rather than bone marrow–resident long-lived plasma cells (LLPCs). LLPCs, which produce the majority of circulating antibodies, express negligible levels of CD20 and thus are not targets of rituximab treatment (28). This is confirmed by commonly unchanged serum Ig levels and sustained vaccine-specific titers after treatment (26, 29, 30). Accordingly, we proposed a speculative model in which an autoreactive fraction of memory B cells and circulating short-lived plasmablasts are responsible for much of the MuSK autoantibody production (3, 31) and recently demonstrated that circulating plasmablasts do indeed contribute to MuSK MG autoantibody production (32).

Given the accessibility of circulating autoantibody-producing cells, we adapted a previously reported approach (33) to produce a high-avidity, fluorescently tagged MuSK tetramer that could identify and assist with sorting rare autoantibody-expressing B cells from patient-derived blood samples. The specificity of the isolation was validated by single-cell sorting of antigen-labeled B cells and by reconstruction of recombinant human mAbs, which were then tested for binding to MuSK.

## Results

### Study subjects.

Patients (*n* = 6, all female; mean age 44 ± 12 years, range 37–63; consistent with reported demographics of MuSK MG in refs. 34–36) with laboratory and clinically confirmed MuSK autoantibody–positive MG were selected for study. Their clinical severity and serum autoantibody status at the time of sampling are summarized in Table 1. The controls included 2 healthy individuals, 1 male aged 37 years and 1 female aged 30 years (Table 1). Both had no history of autoimmune disease and no recent inflammatory events and were negative for serum MuSK autoantibodies.

### Generation of a multimeric, fluorescent MuSK antigen.

We expressed the extracellular domain of MuSK, which was tagged, at the C-terminus, with a BirA site that allows for posttranslational biotinylation. The addition of allophycocyanin-conjugated (APC-conjugated) streptavidin was then used to generate a fluorescent antigen tetramer/multimer (Supplemental Figure 1A; supplemental material available online with this article; https://doi.org/10.1172/jci.insight.127167DS1). To validate that MuSK-specific antibodies were able to recognize the tetramer, antibody binding was tested in a flow cytometry–based assay. Flow cytometry beads, coated with anti–mouse Ig antibodies, were incubated with either the hybridoma-derived murine mAb, 4A3, that recognizes human MuSK or a control mAb, 8-18C5, that recognizes human myelin oligodendrocyte glycoprotein (MOG). Antibody-coated beads were incubated with fluorescent MuSK multimers and then analyzed by flow cytometry. The MuSK multimer was bound by beads that were coated with the MuSK-specific mAb but not those coated with the MOG mAb (Supplemental Figure 1B). These data established that the multimerized, labeled MuSK retained properties required for antibody recognition and was suitable for identifying B cells expressing MuSK-specific B cell receptors. Thus, the reagent was applied for the identification and isolation of human MuSK-specific B cells. Antibody-secreting cells (plasmablast-like phenotype) and antigen-experienced B cells (memory-like phenotype) that bound the fluorescent MuSK multimer (Supplemental Figure 2) were isolated.

### Screening of recombinant human mAbs.

**Figure 1:**
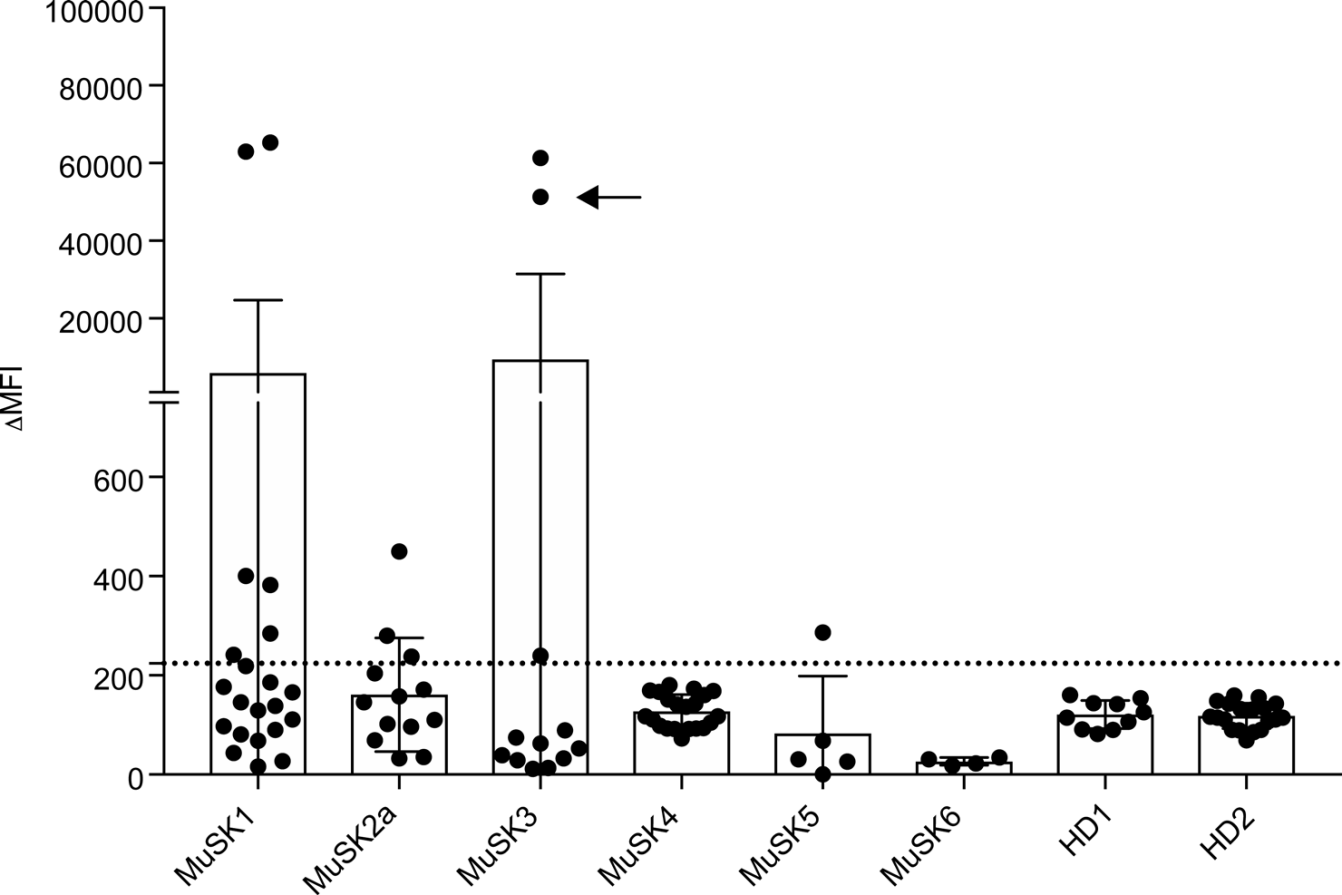
Screening of human recombinant mAbs. Recombinant mAbs were produced from single MuSK multimer-sorted B cells. Binding of these clones to MuSK-expressing cells was determined using a flow cytometry–based antibody-binding assay. Each data point represents the mean ΔMFI of each mAb tested at 1 μg/mL in triplicate. Bars represent the mean of means and error bars the SDs. The mAbs were derived from patients with MuSK MG and healthy donors (HDs): MuSK1 (*n* = 22), MuSK2a (*n* = 5), MuSK3 (*n* = 12), MuSK4 (*n* = 13), MuSK5 (*n* = 21), MuSK6 (*n* = 6), HD1 (*n* = 10), and HD2 (*n* = 19). A human recombinant MuSK mAb that we previously produced from single-cell–sorted plasmablasts (indicated with an arrow) was included with those tested from patient MuSK3. Values greater than the mean plus 4 SDs of the HD-derived mAbs (indicated by the horizontal dotted line) were considered positive.

We cloned and expressed human recombinant mAbs from single sorted memory B cells or plasmablasts, which were positive for staining with the fluorescent MuSK multimer. We cloned 77 mAbs from the 6 MuSK MG patients and another 29 from the 2 healthy controls (Supplemental Table 1). For initial screening of MuSK-binding capacity, all the variable heavy chain domains were cloned into a human IgG1 subclass expression vector, irrespective of their native isotype or IgG subclass usage, which was not determined at this step. The variable light chain domains were cloned into either a κ or λ expression vector based on their native usage. We first screened the mAbs for MuSK binding at 10 μg/mL using a live cell-based assay (CBA). At this mAb concentration, many of the mAbs, including those from the healthy controls, showed binding (not shown). However, using a concentration of 1 μg/mL, 3 mAbs (MuSK1A, MuSK1B, and MuSK3B) from 2 patients (MuSK1 and MuSK3), unlike the mAbs from the 2 healthy donors (HDs), demonstrated robust binding to MuSK (Figure 1 and Table 2). Most other mAbs from the MuSK patients (MuSK2a, MuSK4, MuSK5, and MuSK6) did not bind at this concentration (Figure 1). Consequently, we focused on the 3 robustly binding mAbs (MuSK1A, MuSK1B, and MuSK3B). We also included an additional MuSK mAb that we had previously isolated (MuSK3-28; ref. 32), without the use of the labeled MuSK multimeric antigen, from subject MuSK3. Although production of this MuSK mAb was reported previously, the native IgG subclass, binding properties, MuSK domain specificity, and pathogenic capacity had not been characterized.

### Cellular origins and binding properties of MuSK-specific recombinant mAbs.

During the MuSK-specific B cell sort, the FACS analyzer marked each cell in the scatter plot and its corresponding position in the 96-well plate (index sorting). After the mAbs were expressed and their MuSK specificity validated on the CBA, the exact position of the cell on the scatter plot was determined and consequently its phenotype was assessed. With this approach, we determined (Table 2) that mAbs MuSK1A and MuSK3B were derived from B cells displaying a memory-like phenotype (CD19^+^CD27^+^CD38^–^) and MuSK1B was derived from a B cell displaying a CD38^+^ plasmablast-like phenotype (CD19^+^CD27^+^CD38^hi^). The mAb MuSK3-28 had been isolated from a single-cell–sorted total plasmablast population (32).

### Isotype, IgG subclass usage, and molecular properties of MuSK-specific recombinant mAbs.

The native isotype and IgG subclass were determined using an additional PCR and sequencing step. The mAb MuSK1A was natively expressed as IgG4, mAb MuSK1B as IgG3, and mAb MuSK3B as IgM (Table 2). The mAb MuSK3-28 was natively expressed using IgG4. B cell receptor sequence analysis of the 4 MuSK mAbs revealed that these autoantibodies are represented by diverse clones that use different variable region gene segments (Table 2). A number of somatic mutations, a hallmark of affinity maturation, had accumulated in the variable heavy and variable light CDR regions of the 3 IgG isotype mAbs, strongly suggesting that antigenic selection had occurred. The fourth mAb, which used IgM, did not include any somatic hypermutations. These Ig sequencing data show that MuSK autoantibodies are mostly class switched and suggest that the development of MuSK autoantibodies often requires the processes of clonal selection, affinity maturation, and class switching.

### Binding properties of MuSK-specific recombinant mAbs.

We next sought to examine the binding properties of the IgG mAbs. The IgM isotype–derived mAb, MuSK3B, was not further investigated in this study because this isotype has not been implicated in MuSK MG pathology. Given the importance of IgG4 autoantibodies in MuSK MG and to discount any influence of the subclass constant region on the IgG4 mAbs, we subcloned the variable region of the IgG4 subclass autoantibodies (MuSK1A and MuSK3-28) into human IgG4 expression vectors, thus, matching the native subclass and ensuring they were expressed as divalent, monospecific recombinant mAbs. Unless otherwise noted, the IgG4 subclass versions of mAbs MuSK1A and MuSK3-28 were used.

**Figure 2:**
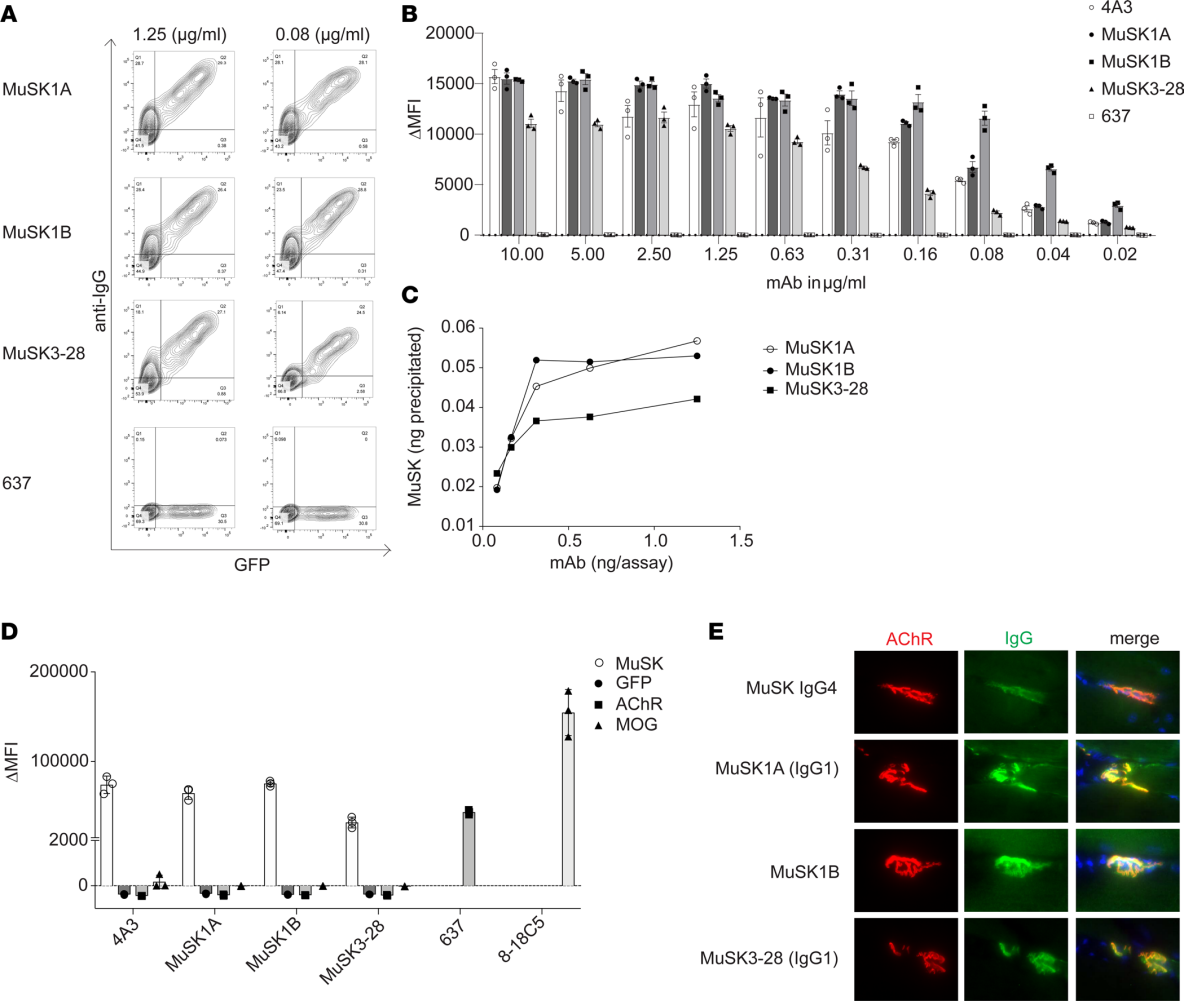
Characterization of human MuSK mAb–binding properties. Binding properties of mAbs MuSK1A, MuSK1B, and MuSK3-28 were tested in several in vitro antibody-binding assays. (**A**) Representative cell-based assay (CBA) flow cytometry plots are shown for 3 MuSK mAbs and a negative control (AChR-specific mAb 637). Binding was tested at both 1.25 and 0.08 μg/mL. The *x* axis represents GFP fluorescence intensity and, consequently, the fraction of transfected HEK cells. The *y* axis represents Alexa Fluor 647 fluorescence intensity, which corresponds to secondary anti–human IgG antibody binding and, consequently, primary antibody binding to MuSK. Hence, transfected cells are located in the right quadrants and transfected cells with MuSK autoantibody binding in the upper right quadrant. (**B**) Binding to MuSK was tested over a wide range of mAb concentrations in the CBA. Controls included the MuSK-specific humanized mAb 4A3 and AChR-specific mAb 637 tested with MuSK mAbs MuSK1A, MuSK1B, and MuSK3-28. Each data point represents a separate replicate within the same experiment. Bars represent means and error bars SDs. (**C**) A solution phase radioimmunoassay was used to measure MuSK binding over a range of mAb concentrations. Each data point represents a value within the same experiment. (**D**) Specificity of the mAbs was evaluated using CBAs that tested binding to HEK cells transfected with MuSK, GFP alone, AChR, or MOG. Positive controls included MuSK-specific humanized mAb 4A3, AChR-specific mAb 637, and MOG-specific 8-18C5. Each data point represents a separate replicate within the same experiment. Bars represent means and error bars SDs. (**E**) Immunofluorescent staining of mouse NMJs. Tibialis anterior muscles were cut longitudinally in cryosections and fixed with PFA. AChRs were stained with Alexa Fluor 648 α-bungarotoxin (shown in red) and DNA with Hoechst (shown in blue in the merged panels). The first row shows staining with polyclonal IgG4 from a patient with MuSK MG. Binding of mAbs (MuSK1A, MuSK1B, MuSK3-28) against MuSK (1.6 μg/mL for 1 hour) was detected with goat anti–human IgG Alexa Fluor 488 (IgG, shown in green). In **A**–**E** the IgG4 subclass mAbs MuSK1A and MuSK3-28 were tested in their native IgG subclass unless indicated otherwise.

Binding of the mAbs was tested over a range of concentrations using a live CBA. These tests demonstrated that binding could be detected with only 20 ng/mL for the IgG mAbs MuSK1A, MuSK1B, and MuSK3-28 (Figure 2, A and B). An independent RIPA, commonly used for clinical diagnosis of MuSK MG, showed that as little as 0.3 ng of mAbs MuSK1A, MuSK1B, and MuSK3-28 could bind 30% to 50% of ^125^I-MuSK (approximately 1 fmol/assay; Figure 2C). To explore their specificity and potential pathogenicity, we also tested these mAbs in CBAs using GFP-transfected HEK cells or cells transfected with AChR or MOG (Figure 2D). MOG was chosen because its structure (37), a classical Ig (Ig variable domain) fold, is highly similar to that of MuSK (38). The mAbs MuSK1A, MuSK1B, and MuSK3-28 did not show any detectable binding to GFP-transfected HEK cells or HEK cells expressing AChR or MOG on their surface (Figure 2D). Finally, we tested the mAbs on sections of mouse muscle tissue to determine whether they could recognize mouse MuSK. The mAbs that were highly positive in the CBA, using human MuSK, also bound to mouse NMJs where the mAbs closely colocalized with AChRs (Figure 2E). These data also demonstrated that the mAbs can recognize MuSK when presented in its native biological environment, an important requisite for future pathogenicity experiments in mouse-derived cells and disease models.

### MuSK autoantibody epitope mapping.

**Figure 3:**
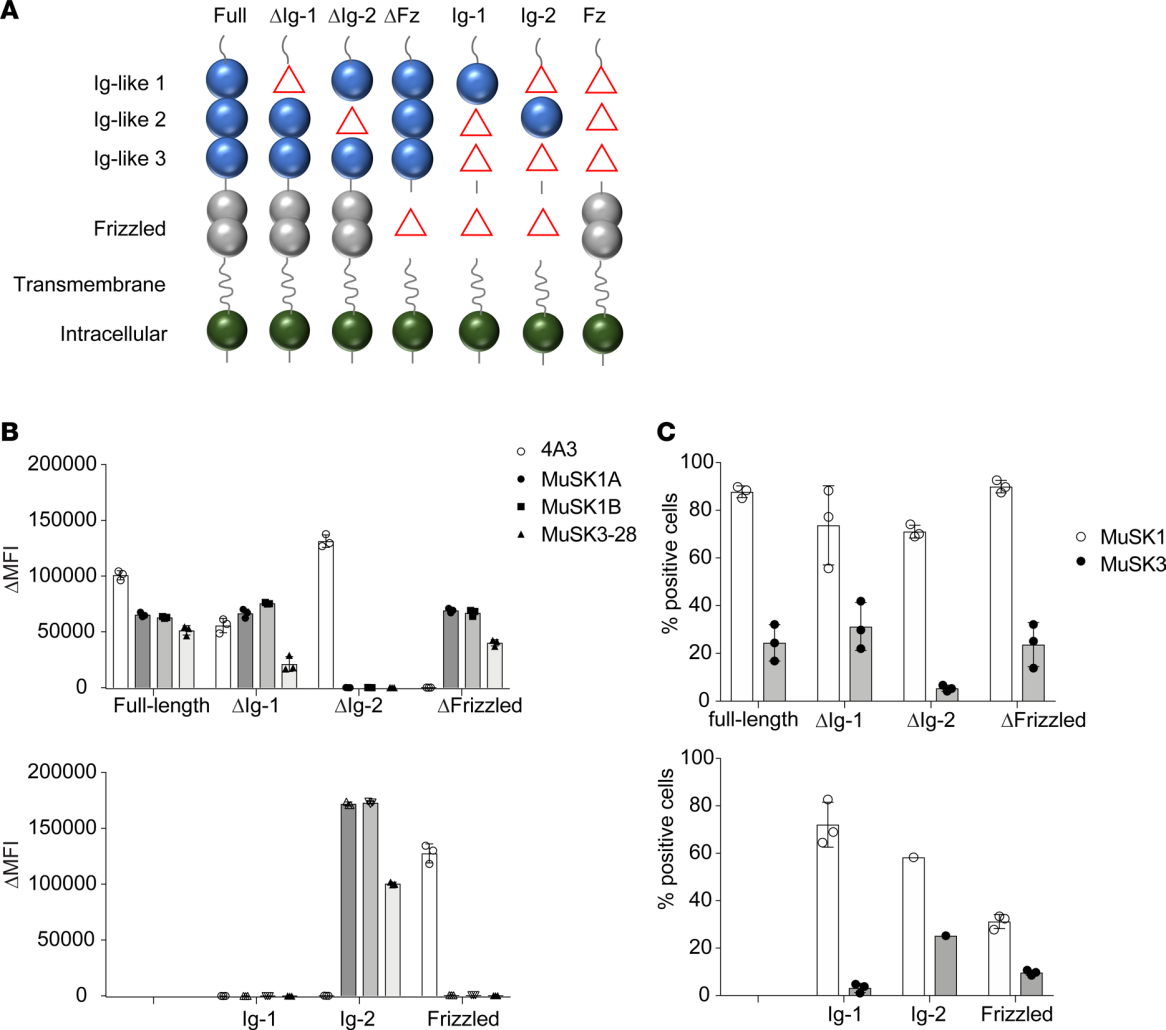
MuSK domain-binding results. To map the human MuSK mAb epitopes, MuSK constructs that had particular domains deleted and full-length MuSK were each expressed in HEK cells and tested with the CBA. (**A**) The schematic illustrates the mutant forms of MuSK. For example, “ΔIg-1” includes only the Ig-like domains 2 and 3 and the frizzled-like (Fz-like) domain because the Ig-like domain 1 was deleted (shown as “Δ” in the schematic). Similarly, “Ig-1” includes only the Ig-like domain 1 because the Ig-like domains 2 and 3 and Fz-like domain were deleted (shown as “Δ” in the schematic). Binding of mAbs (MuSK1A, MuSK1B, MuSK3-28, and the positive control humanized MuSK mAb 4A3) to these mutant forms of MuSK was tested in our standardized flow cytometry CBA. Results for each (**B**) mAb or (**C**) serum specimen are shown. Serum was obtained from the same patients from whom the mAbs were derived. Each data point represents a separate replicate within the same experiment. Bars represent means and error bars SDs.

To map the targets of the mAbs, we engineered a series of plasmid constructs (Figure 3A and Supplemental Figure 3) to express either individual subdomains of MuSK or MuSK with deletions of individual subdomains and tested binding of the mAbs using a CBA. The mAbs MuSK1A, MuSK1B, and MuSK3-28 bound to HEK cells expressing full-length MuSK, MuSK ΔIg1, MuSK Δfrizzled (ΔFz), and MuSK Ig2 only (ΔIg1, Ig3, and Fz; Figure 3B) but did not bind to MuSK in which the Ig-like domain 2 was deleted (ΔIg2) or when the Ig-like domain 1/Fz-like domain were expressed alone (Figure 3B). Thus, mAbs MuSK1A, MuSK1B, and MuSK3-28 recognize epitope(s) in Ig-like domain 2. The control humanized mAb 4A3, previously produced (32), recognized the Fz-like domain (Figure 3B).

We also tested sera from MuSK MG patients MuSK1 and MuSK3 for binding to the different MuSK domain constructs. MuSK1 serum contained autoantibodies that recognized full-length MuSK, as well as each of the domain deletion constructs and the isolated domain constructs. These findings indicate that MuSK1 serum contains a heterogeneous collection of autoantibody specificities, which collectively recognize epitopes present in all the tested MuSK domains (Figure 3C). MuSK3 serum displayed lower reactivity compared with MuSK1 serum when testing binding to full-length MuSK. In addition, MuSK3 serum contained autoantibodies that preferentially recognized isolated MuSK domains that included the Ig-like domain 2, indicating that the epitope(s) could be more restricted in this patient (Figure 3C). These results using MuSK1 and MuSK3 serum provide further evidence for surface expression of the constructs tested. Moreover, the results concerning MuSK3 serum binding showed that the specificity of the mAb MuSK3-28 for the MuSK Ig-like domain 2 reflected the specificity of the circulating autoantibody repertoire.

### Pathogenic capacity — MuSK mAbs interfere with agrin-induced AChR clustering.

**Figure 4:**
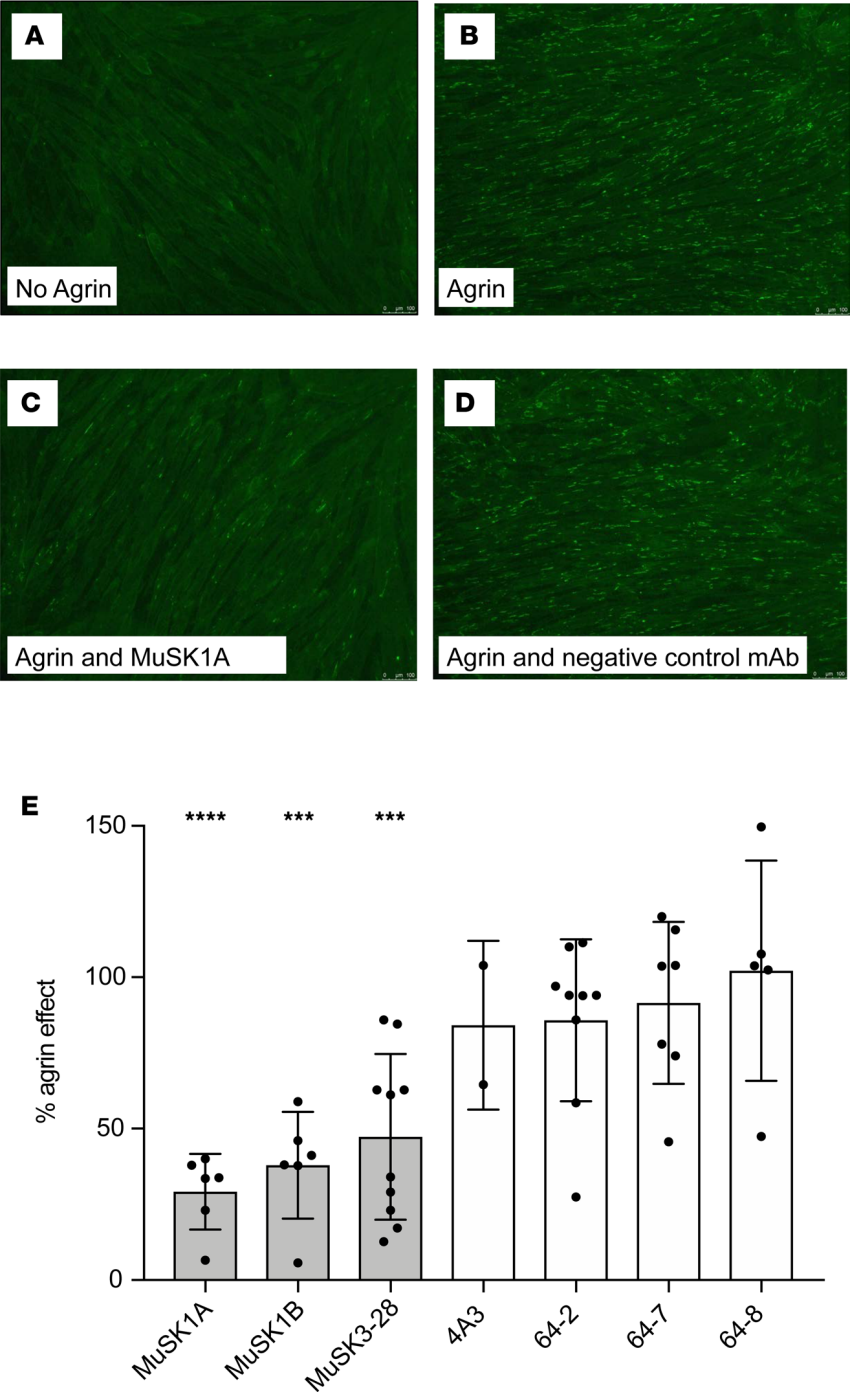
AChR-clustering assay in C2C12 mouse myotubes demonstrates pathogenic capacity of MuSK mAbs. The presence of agrin in C2C12 myotube cultures leads to dense clustering of AChRs that can be readily visualized with fluorescent α-bungarotoxin and quantified. Pathogenic MuSK autoantibodies disrupt this clustering. Three different human MuSK-specific mAbs, the humanized murine control MuSK mAb 4A3, and 3 human non–MuSK-specific mAbs derived from AChR MG patient plasmablasts (plasmablasts 64-2, 64-7, and 64-8) were tested for their ability to disrupt the AChR clustering. Each mAb was added to the cultures at 1 μg/mL. (**A**–**D**) Representative images (original magnification, ×100) from the clustering experiments are shown. (**A**) Cultured myotubes do not show AChR clustering until (**B**) agrin is added (bright spots reveal AChR clusters). (**C**) The mAb MuSK1A added at 1 μg/mL inhibits clustering (**D**), whereas a control mAb does not inhibit the formation of AChR clusters. (**E**) Clustering of AChR was quantified relative to the measured effect of agrin. Quantitative results are normalized to clustering induced by only agrin. Each data point represents the mean value from an independent experiment. Bars represent the mean of means and error bars the SDs. Multiple-comparisons ANOVA (against the pooled results for the 3 human non–MuSK-specific mAbs), Dunnett’s test; *P < 0.05, **P < 0.01, ***P < 0.001, and ****P < 0.0001, shown only when significant.

To evaluate the pathogenicity of the MuSK-specific recombinant mAbs, we used the well-established in vitro C2C12 AChR-clustering assay. The C2C12 mouse myotubes express all the components that are required for agrin to stimulate AChR clustering (39). Serum-derived MuSK autoantibodies have been demonstrated to interrupt this interaction and consequentially inhibit AChR clustering (7, 18, 23). The C2C12 myotubes were incubated with each of the 3 MuSK mAbs and controls; then AChR clusters were visualized (Figure 4, A–D) and the mean number of clusters recorded. All 3 mAbs reduced agrin-induced AChR clustering, whereas mAb 4A3, which recognized the Fz-like domain, had no effect (Figure 4E). These findings indicate that mAbs MuSK1A, MuSK1B, and MuSK3-28 are pathogenic in this model. The ability of the mAbs to induce AChR clustering in the absence of agrin was also evaluated. When added to C2C12 cultures, each of the 3 MuSK mAbs induced a modest, although not significant, increase in AChR clustering (Supplemental Figure 4) compared with 3 non–MuSK-binding mAbs and the MuSK Fz-like domain-specific 4A3 mAb.

### Pathogenic capacity — MuSK mAbs modify agrin-induced MuSK phosphorylation.

**Figure 5:**
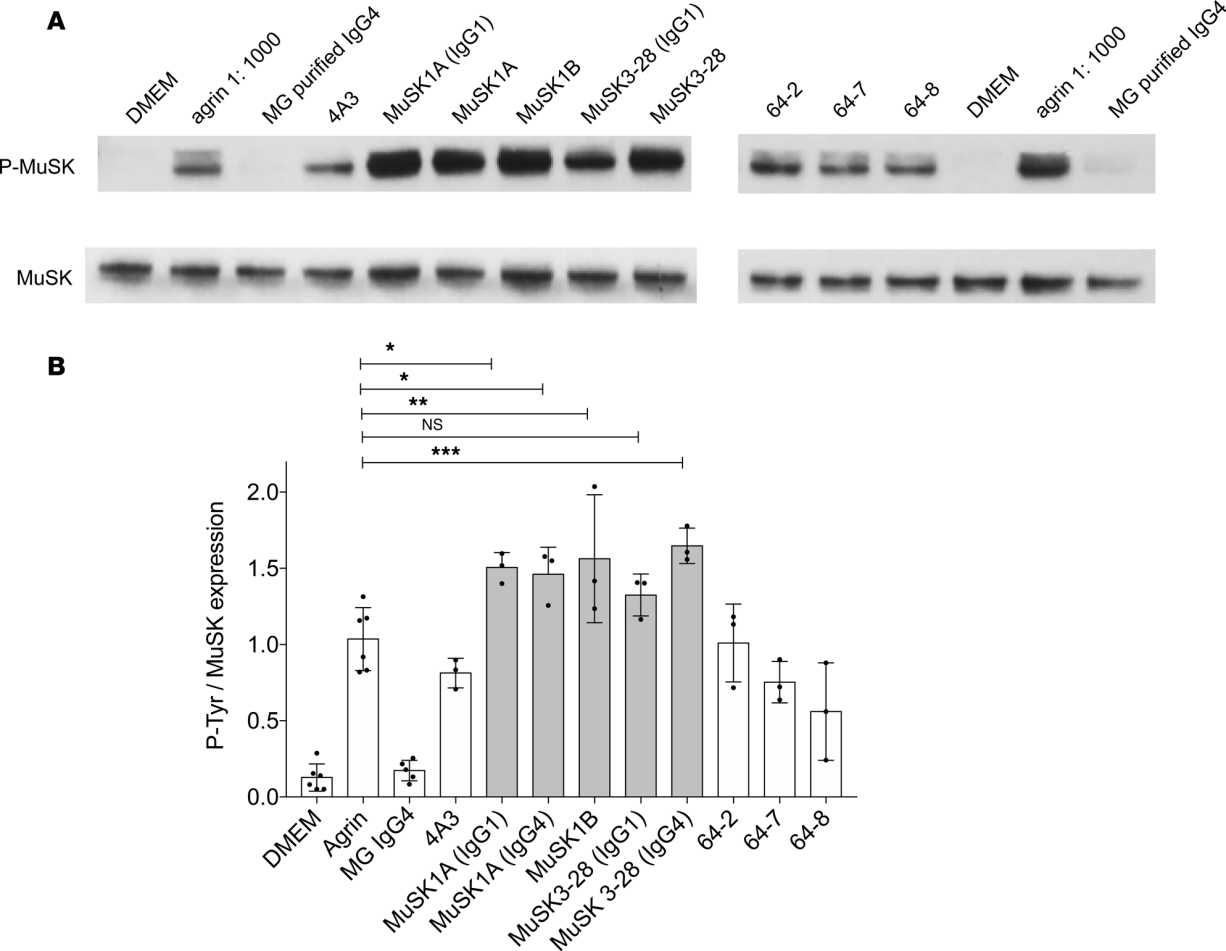
MuSK mAbs can amplify agrin-induced tyrosine phosphorylation. (**A**) Immunoblots showing phosphotyrosine bands and related MuSK expression in C2C12 murine myotubes that were incubated with agrin in the presence of MuSK MG serum-derived IgG4 or recombinant MuSK/control mAbs. 4A3 is a humanized murine MuSK mAb; MuSK1A, MuSK1B, and MuSK3-28 are human MuSK mAbs from patients with MuSK MG; and 64-2, 64-7, and 64-8 are non–MuSK-binding human mAbs derived from AChR MG patient plasmablasts. IgG4 subclass mAbs MuSK1A and MuSK3-28 were expressed in vectors reflecting the native subclass and as IgG1 (as indicated). (**B**) Normalized densitometry analysis results from the MuSK phosphorylation immunoblots are plotted. Each data point represents an independent experiment. Bars represent means and error bars SDs. Phosphorylation of MuSK was determined by normalizing to MuSK expression, detected by a commercial anti-MuSK antibody after stripping the blot, and the ratio of phosphotyrosine MuSK/MuSK is plotted. Multiple-comparisons ANOVA (versus agrin), Dunnett’s test; ns *P* > 0.05, *P < 0.05, **P < 0.01, and ***P < 0.001, shown for MuSK mAbs versus agrin comparisons.

One of the crucial steps in the agrin/LRP4/MuSK/DOK7 pathway is MuSK phosphorylation. Serum IgG or IgG4 antibodies from patients with MuSK MG inhibit agrin-induced MuSK tyrosine phosphorylation (20). MuSK MG serum-derived IgG or recombinant human mAbs were added to cultured C2C12 myotubes, with agrin. MuSK tyrosine phosphorylation was then detected using immunoblotting with a phosphotyrosine-specific antibody (Figure 5A). Agrin-induced phosphorylation was blocked by the patient-derived serum IgG whereas the 3 non–MuSK-binding mAbs and the control mAb 4A3, which recognizes an epitope in the Fz-like domain, did not alter the agrin-induced MuSK phosphorylation (Figure 5B). By contrast, and intriguingly, the 3 MG patient–derived MuSK-specific mAbs, MuSK1A, MuSK1B, and MuSK3-28, all modestly amplified the agrin-induced phosphorylation (Figure 5B). Thus, these mAbs increased agrin-induced MuSK phosphorylation while inhibiting agrin-induced AChR clustering. The mAbs MuSK1A and MuSK3-28 were tested both as their native IgG4 subclass and as IgG1. Similar amplification of agrin-induced phosphorylation was observed with both subclasses (Figure 5B). These results suggest that divalent (and monospecific) MuSK autoantibodies that bind Ig-like domain 2 can activate MuSK phosphorylation, irrespective of their subclass.

## Discussion

There is much interest in production of human mAbs from patients with antigen-specific autoimmune diseases, but identification and isolation of autoantibody-producing cells is challenging because many reside in the lymphatics, bone marrow, or other tissue compartments and are scarce in the circulation (33, 40). Moreover, even if circulating antigen-specific B cells can be detected (41, 42), screening approaches, such as Epstein-Barr virus transformation of circulating B cells or bulk isolation of antigen-experienced B cells, may provide only low yields of the relevant antibodies (43–45). Here, we used a multimerized, fluorescent MuSK construct to identify, capture, and characterize mAbs. Three mAbs from 2 patients with MG bound at sub-nanomolar concentrations to MuSK. These autoantibodies, when expressed as divalent IgG1 or IgG4 subclasses, bound to MuSK at the NMJ, colocalizing with AChRs, and demonstrated the pathogenic mechanism of inhibition of agrin-induced AChR clustering, typical of the patients’ serum autoantibodies. Surprisingly, MuSK phosphorylation, which is typically inhibited by patients’ serum or native monovalent IgG4 autoantibodies, was moderately increased rather than reduced by the mAbs. This interesting finding is likely due to the divalence of the expressed MuSK mAbs.

A large number of MuSK autoantibodies were identified on the first screens when testing the mAbs at 10 μg/mL, but many showed considerably lower or absent binding capacity at 1 μg/mL. Many of these mAbs were derived from IgM B cells of HDs and patients, had low or absent somatic mutational loads, and are, therefore, unlikely to have been antigen driven and thus may be of inconsequential biological significance in MG. It is possible that these IgM-expressing B cells are part of a normal repertoire that can bind multivalently but nonspecifically to the tetrameric MuSK protein. Indeed, sensitive and specific detection of MuSK autoantibodies using CBAs on clinical samples requires the use of IgG-Fc–specific antisera because of the risk of detecting nonspecific IgM binding to MuSK (46). A similar problem is seen with testing autoantibodies against MOG and may reflect interactions between IgM molecules and the extracellular Ig-like domains of these proteins (47).

A set of mAbs, which did demonstrate high binding capacity to MuSK, were studied further. The extracellular domain of MuSK is composed of 3 Ig-like domains (Ig1, -2, and -3) and a cysteine-rich Fz-like domain, which occupies the region between the Ig-like domains and the extracellular juxtamembrane region (38, 48). The majority of patient serum–derived MuSK autoantibodies are reported to recognize the N-terminal Ig-like domains Ig1 and Ig2 (7, 14); Ig1 interacts with LRP4 and is thought to form the most pathophysiologically relevant epitope because of inhibition of LRP4 binding (20, 49). It was surprising, therefore, that all 3 IgG mAbs recognized the Ig2 domain of MuSK. Two of the 3 were isolated with the tetrameric antigen, and it is possible that this conformation preferentially selects for Ig-like 2 domain autoantibodies. However, because the biotinylation site was in the C-terminal of the extracellular domain, this seems less likely, and the Ig-like 2 domain specificity may simply represent a stochastic or patient-specific event. The humanized murine control MuSK mAb 4A3, by contrast, recognized the Fz-like domain. This domain in mammalian MuSK appears to be dispensable for AChR clustering (50, 51), and mAb 4A3 provided a useful control for the experimental studies discussed below.

In patients, the majority of MuSK autoantibodies are IgG4, while a variable minority are IgG1, -2, or -3 (14, 52). IgG4 antibodies have very weak inter–heavy chain S-S bonds and are able to exchange Fab arms with other IgG4s; thus, IgG4 antibodies become functionally monovalent (24). MG patient serum antibodies have been shown to be largely Fab arm exchanged, and serum MuSK autoantibodies reduce AChR clustering in the C2C12 model (23), as do polyclonal Fabs against MuSK (21). However, because our IgG4 mAbs were expressed as individual clones, they were not able to Fab arm exchange with other IgG4s and were thus divalent. Both MuSK1A and MuSK3-28 are of the IgG4 subclass, while MuSK1B is IgG3 subclass. However, irrespective of their native subclass or the Ig-like domain targeted by the mAbs, all 3 IgG mAbs inhibited the formation of agrin-induced AChR clusters in the C2C12 myotube model, which suggests that they are likely to have full pathogenic potential and questions the pathogenic dominance of monovalent IgG4 autoantibodies against the Ig-like 1 domain in MuSK MG. Indeed, a previous study (21) demonstrated that IgG1, -2, and -3 MuSK autoantibodies purified from 2 patients with MuSK MG, which did not inhibit LRP4 binding to MuSK, inhibited AChR clustering even more effectively than IgG4 MuSK autoantibodies from those patients. Overall, the results of these highly selected MuSK mAbs support that the patients’ autoantibodies, of all IgG subclasses, are capable of inhibiting MuSK function and do not per se require one to invoke other mechanisms. Nevertheless, in contrast with native IgG4, both the native IgG1 and IgG3 subclasses are effective at initiating complement activation. Consequently, the presence of IgG3 MuSK mAbs in 1 patient, along with several recently reported IgG1 MuSK mAbs (53), suggests that the immunopathology of MuSK MG may include inflammatory, potentially damaging, mechanisms as well as inhibitory mechanisms. As previously proposed, IgG-mediated damage may be most important in isolated muscles, such as facial and bulbar muscles that are susceptible to atrophy (21).

When agrin binds to LRP4, which then forms a complex with MuSK, it leads to MuSK autophosphorylation, recruitment of DOK7, and eventually rapsyn and AChR clustering, as usually studied in the C2C12 myotubes used here. Until recently, inhibition of phosphorylation was considered in determining the pathogenicity of MuSK autoantibodies. However, the results here question this assumption. All 3 human MuSK mAbs inhibited AChR clustering in the C2C12 cells, but these divalent autoantibodies led to modest but significant increases in phosphorylation rather than reduced phosphorylation. The explanation probably lies in their divalence, which may have been associated with the cross-linking of MuSK as found in murine models (19, 51, 54). The native monovalent IgG4 mAbs would not cross-link MuSK or increase phosphorylation, as recently shown in cloned IgG4 mAbs (53). Patient MuSK IgG1, -2, and -3 autoantibodies act in a similar manner, inhibiting AChR clustering while not inhibiting MuSK phosphorylation (MC, DB, and AV, unpublished observations). The mAbs described here will be essential for understanding how the divalent IgG1, -2, or -3 MuSK autoantibodies cause inhibition of AChR clustering and alter MuSK phosphorylation and studying more fully their contribution to the disease.

The mAb MuSK1B was derived from a cell with a plasmablast-like phenotype, expressing both CD27 and high levels of CD38. This plasmablast, along with that which produced mAb MuSK3-28 (32), supports the notion that this circulating, short-lived cell type contributes to MuSK MG immunopathology (31). The isolated cell that yielded mAb MuSK1A displayed a memory B cell–like phenotype. The appearance of this cellular subtype among MuSK autoantibody-expressing cells suggests that immunological memory might have been established and that these cells can provide a reservoir from which autoantibody-secreting plasmablasts originate. Given that memory B cells express CD20, their direct elimination by CD20-targeted therapy (rituximab) may be the mechanism by which this treatment induces remarkable serum autoantibody decline and excellent clinical response in patients. That memory B cells do not secrete antibodies leaves open the possibility that such cells play an antibody-independent role in the immunopathology. Antigen presentation by B cells contributes to autoimmunity (55–57) and is necessary for models of neurological autoimmunity (58, 59). Indeed, the autoimmune mechanism in neuromyelitis optica, although principally attributed to aquaporin-4 autoantibodies, is accompanied by B cell–mediated antigen presentation to T cells and cytokine production of both the proinflammatory and antiinflammatory varieties associated with a more complex neuroinflammation (60).

Three mAbs that bound to MuSK robustly were successfully isolated from 2 patients, while 3 additional patient samples did not yield any strong MuSK-binding mAbs. These results suggest that such cells may be rare in the circulation of MG patients, even though such patients were experiencing active disease and had conspicuous serum autoantibody titers at the time of specimen collection. Furthermore, the clinical status of the patients may be important for isolation of the disease-specific cells. The patients in this study, from whom the strong-binding mAbs were sourced, were experiencing a disease exacerbation after having achieved remission through B cell depletion therapy. Remission often affords the withdrawal of treatment, including immunomodulatory treatments, that may suppress activated B cells and plasmablasts. Indeed, elevated plasmablasts have been associated with autoimmune disease activity (61). Thus, these data support the concept that breakthrough relapses after withdrawal of aggressive immunomodulatory treatments that previously had successfully suppressed the immune response could be the optimal time at which to study the otherwise sequestered pathogenic B cells and plasmablasts.

The collective data in this report highlight the importance of demonstrating the full spectrum of autoantibody characteristics and pathological potential. We anticipate that these human MuSK mAbs and the approach to their isolation will be recognized as highly valuable tools in future studies. First, these mAbs can be used to dissect the molecular mechanisms of MuSK autoantibody pathology, particularly for understanding how MuSK phosphorylation can be associated with inhibition of clustering, using both in vitro and in vivo models. Second, once the relevance of the different IgGs and epitope specificities are established, the development of preclinical models that do not rely on the limited human MG-derived serum autoantibodies will aid in the investigation of MuSK immunopathology and help explain why facial and bulbar muscle groups are the principal target in patients. Third, the identification and isolation of rare MuSK mAb–producing cells, using the fluorescent MuSK antigen, will allow further investigation into their roles in initiation and perpetuation of the disease and whether their frequency in the circulation may represent a valuable biomarker for predicting relapse and therapeutic response. Finally, these cells should be viable targets for antigen-targeted therapies (62) that would seek to eliminate only those cells that directly contribute to autoimmunity, which would replace current nonselective immune-modulating treatments.

## Methods

### Isolation of serum and PBMCs from patients with MuSK MG.

Peripheral blood samples were obtained from 2 healthy donors (HD1 and HD2) and 6 patients (MuSK1–6) with autoantibody and clinically confirmed MuSK MG. Patients showed typical clinical and serological features of MuSK MG (Table 1). PBMCs were isolated by Ficoll’s separation and stored in liquid nitrogen until use, using a described protocol (63). Time-locked serum specimens were also obtained.

### MuSK multimer generation.

The extracellular domain of human MuSK was subcloned into the pMT/Bip/His-A vector. The C-terminal region contained a short, flexible linker followed by a BirA site (amino acids: GLNDIFEAQKIEWHE), downstream of which was a thrombin-cleavable (amino acids: LVPRGS) 6× histidine tag. Protein expression was induced in S2 *Drosophila* cells (Thermo Fisher Scientific). Culture supernatant was collected and MuSK protein was purified using cobalt resin beads (Thermo Fisher Scientific) according to the manufacturer’s instructions. For tetramer formation, MuSK protein was biotinylated by incubation with BirA enzyme at a 1:100 molar ratio overnight at 4°C in a buffer containing 50 mM Tris, 50 mM bicine at pH 8.3, 10 mM magnesium acetate, 10 mM adenosine-5’-triphosphate, and 50 μM biotin. Excess biotin was removed using a 10-kDa MWCO Slide-A-Lyzer dialysis cassette (Thermo Fisher Scientific). Fluorescent multimers were formed using stepwise addition of APC-conjugated streptavidin (Invitrogen) to biotinylated MuSK until a 1:4 molar ratio was reached.

### Flow cytometry and cell sorting.

For sorting MuSK multimer–reactive B cells, B cells were enriched from cryopreserved PBMCs using negative selection beads (Stemcell Technologies). They were incubated with live/dead stain, then stained with 20 μg/ml MuSK multimer on ice for 30 minutes. Cells were then costained (using manufacturer’s recommended dilutions) with fluorescently labeled antibodies against CD3 (Invitrogen, Pacific orange; UCTH1), CD14 (Invitrogen, Pacific orange; TUK4), CD19 (BD Biosciences, PE Cy7; SJ25C1), CD27 (BD Biosciences, PE; M-T271), and CD38 (BD Biosciences, V450; HB7) before sorting on an FACSAria (BD Biosciences) instrument. For general B cell immunophenotyping, B cells were defined as live CD3^–^CD14^–^CD19^+^ cells, memory B cells as live CD3^–^CD14^–^CD19^+^CD27^+^CD38^–^ cells, and antibody-secreting cells (plasmablast phenotype) as CD3^–^CD14^–^CD19^+^CD27^+^CD38^hi^.

### MuSK, AChR, and MOG mAbs.

A set of mAbs for binding to MuSK, AChR, and MOG were used as controls. Cell culture supernatant from either an established murine MuSK mAb (4A3; ref. 32) or a murine MOG mAb (8-18C5; ref. 64) hybridoma was applied to a Protein G/Sepharose column (GE Healthcare) to isolate the IgG. We also engineered the MuSK mAb (4A3) and the MOG mAb (8-18C5) as chimeric mouse-human recombinant mAbs. They were produced to contain the murine mAb heavy and light chain variable regions fused to the respective human constant regions using an approach we described (65). These chimeric recombinant mAbs served as positive controls in the human antibody–binding assays and did not require a substitute (murine-specific) secondary antibody because the constant regions were identical to those of human mAbs. The AChR mAb (clone 637) was derived from a human MG thymus (24, 25, 66). The variable regions were synthesized as gBlock gene fragments (Integrated DNA Technologies), then subcloned into expression vectors, expressed, and purified using approaches we described (67).

### Recombinant human mAb production, IgG subclass determination, and subcloning.

Detailed methods describing the recombinant human mAb production are available in Supplemental Methods. Briefly, reverse transcription of fresh or frozen single-cell–sorted, antigen-labeled B cell RNA; nested PCR reactions; subcloning into IgG expression vectors; antibody expression; and purification were all performed as described (63). To determine the isotype and IgG subclass, a specialized single-cell PCR using leftover cDNA from the same single cells used to make individual mAbs was performed. This PCR used a primer in a downstream region of the IgG such that the PCR product included a region of the IgG1, -2, -3, and -4 that is unique to each subclass (68, 69). Thus, identification of IgG subclass required sequence alignment of this region to each of the 4 human IgG subclasses. Following the determination of the endogenous IgG subclass for each human mAb, the variable heavy chain region was subcloned into the respective subclass-containing expression vectors.

### Cell-based antibody assays.

Antibody binding was tested using live HEK293T cells (ATCC, CRL-11268) transiently transfected with DNA encoding MuSK, AChR with rapsyn, or MOG (all coding human proteins), using an assay protocol we described (32). The following plasmid constructs were used for expression: full-length human MuSK (21) subcloned into a p-IRES2–EGFP vector, which delivered translation of MuSK and GFP separately; human AChR α-, β-, δ-, or ε-subunits subcloned into pcDNA3.1-hygro plasmid vectors (70) and rapsyn (70), which was subcloned into a p-EGFP–N plasmid (Clontech Laboratories); full-length human MOG (47) was also subcloned, like rapsyn, into the pEGFP-N plasmid, which produced rapsyn or MOG as fusion proteins with C-terminal GFP. CBA results were calculated as ΔMFI and percentage of transfected cells that bound secondary antibody (termed “% positive”) as follows: (a) ΔMFI = Alexa Fluor 647 MFI in MuSK GFP–transfected cells minus Alexa Fluor 647 MFI in untransfected cells of the same tube; (b) % positive cells = % cells in upper right quadrant divided by % cells in upper right and lower right quadrants.

### MuSK protein mutagenesis.

Human MuSK deletional mutants were generated by modifying the full-length MuSK expression construct (Supplemental Figure 3). Regions coding for specific domains were deleted using the Q5 Site-Directed Mutagenesis Kit (BioLabs) according to the manufacturer’s instructions. Primer sequences were generated using the NEBaseChanger tool. All construct modifications were confirmed through Sanger-based DNA sequencing.

### Ig sequence analysis.

The heavy- and light-chain variable region germline gene segments were identified with the IMGT/V-QUEST program (71) version 3.4.14 (10 September 2018) — IMGT/V-QUEST reference directory release: 201839-3 (September 26, 2018). Somatic mutations resulting in replacement amino acids were evaluated through the alignment to germline genes provided by the IMGT/V-QUEST program. Ig isotype and IgG subclasses were determined by aligning acquired sequences to those present in the ImMunoGeneTics repertoire reference set (72).

### Immunofluorescence mouse muscle sections.

The binding of the different human mAbs was analyzed by immunofluorescence using cryosections of mouse tibialis anterior muscles (obtained from the Central Animal Testing Facilities of Maastricht University). Muscles were cut longitudinally at 10-μm thickness on a Leica CM3050 S cryostat; sections were mounted on gelatin-coated glass slides and stored at –80°C. After thawing, cryosections were fixed with 4% paraformaldehyde (PFA, Sigma-Aldrich) for 10 minutes at room temperature and then blocked for 30 minutes with 2% bovine serum albumin (GE Healthcare). Sections were incubated for 1 hour at room temperature with 1 of the different human mAbs (1.5 μg/mL each) combined with Alexa Fluor 647–conjugated α-bungarotoxin (1:300, B35450, Thermo Fischer Scientific). As controls, protein G–purified IgG from a patient with MuSK MG (final concentration 5 μg/mL) was used instead of the mAbs. After washing, slides were incubated with human Fc-γ–specific Alexa Fluor 488–conjugated goat F(ab′)_2_ (3 μg/mL, 109-546-170, Jackson ImmunoResearch), combined with Alexa Fluor 594–conjugated streptavidin (1:20,000, S11227, Invitrogen) and Hoechst 33342 (2 μg/mL, B2261, MilliporeSigma) for 1 hour at room temperature in the dark. Sections were washed and mounted with 80% glycerol. All washing steps consisted of 3 incubations of the slides (5 minutes at room temperature) in 0.05% Triton X-100. Endplates were identified using the red immunofluorescence of the α-bungarotoxin staining. Triple-fluorescent photomicrographs of the endplate regions were acquired using μManager software ver2.0 (73) on an Olympus BX51WI spinning-disk confocal fluorescence microscope with a Hamamatsu EM-CCD C9100 digital camera. Endplates were analyzed using ImageJ software (NIH) as described (73, 74). All staining procedures and fluorescent analysis were performed on coded samples by 2 independent, blinded investigators.

### AChR-clustering assay.

The AChR-clustering assay was performed as described (7). Briefly, C2C12 mouse myoblasts (ATCC) were grown in DMEM supplemented with 20% fetal bovine serum (FBS) and 1% penicillin/streptomycin (Gibco). C2C12 cells were plated in 24-well plates and differentiated with DMEM supplemented with 2% FBS, 0.5% penicillin/streptomycin, and 1 μM insulin (MilliporeSigma). As soon as fusion was evident (36–48 hours), AChR clustering was induced for 14 hours with 10 ng/mL (0.1 nM) agrin (R&D Systems). mAbs were applied at 1 μg/mL with agrin or alone. After the induction of AChR clustering, AChRs were visualized through the application of 1 μg/mL Alexa Fluor 647–labeled α-bungarotoxin (Invitrogen) for 1 hour at 37°C. Following staining, cells were washed twice with medium (5 minutes at 37°C) and fixed with 3% PFA for 20 minutes at room temperature. Microscopy was performed at a ×100 magnification on a Leica DMi8 fluorescence microscope. For each well, 4 visual fields of 100% myotube confluence were selected on phase contrast and captured on fluorescence; AChR clusters were counted using ImageJ software. For each condition, duplicate wells were used, and the mean of the clusters per visual field per condition was calculated. Experiments were performed at a minimum of 3 repetitions and were normalized for the effect of agrin. Reported results are from experiments in which a minimum 3-fold effect of agrin-induced clustering over the baseline was observed.

### MuSK tyrosine phosphorylation assays.

Myotubes (C2C12) were stimulated with 0.4 nM neural agrin or agrin with purified serum IgG4 (0.5 nM) from MuSK MG patients or mAbs (1 μg/mL) for 30 minutes at 37°C. To detect and quantify MuSK phosphorylation levels, myotubes were extracted in cold lysis buffer (10 mM Tris-HCl, 1 mM EDTA, 100 mM NaCl, 1% Triton X-100, 1× protease inhibitor cocktail, 1× phosphatase inhibitor cocktail) followed by centrifugation (16,000 *g*). To precipitate endogenous MuSK, the whole-cell lysate was incubated with an anti-MuSK polyclonal antibody (AF562, R&D Systems) at 4°C overnight. Bound antibody was captured with Dynabeads protein G (Invitrogen). Bead-precipitated proteins were eluted into SDS sample buffer, subjected to SDS-PAGE, and incubated with monoclonal mouse anti-phosphotyrosine antibody (4G10, Upstate Biotechnology), which was detected using a goat anti–mouse HRP antibody (P 0447; Dako, Denmark) at 1:1000 dilution. The membrane was then harshly stripped (in 62.5 mM Tris buffer at pH 6.8, containing 2.0% SDS and 0.8% β-mercaptoethanol) and reprobed for MuSK by incubating with a goat anti-MuSK polyclonal antibody (AF562, R&D), which was detected using a polyclonal rabbit anti–goat HRP antibody (P 0449; Dako, Denmark) at 1:1000 dilution. Densitometry of bands was analyzed using ImageJ software and the level of MuSK phosphorylation normalized to levels of immunoprecipitated MuSK.

### Statistics.

AChR clustering on the C2C12 cells and MuSK tyrosine phosphorylation were analyzed using a 1-way ANOVA with Dunnett’s correction. *P* values below 0.05 were considered significant. Statistics were performed on GraphPad Prism (version 7.0a) software.

### Study approval.

This study was approved by the Human Investigation Committee at the Yale School of Medicine. Informed consent was obtained from all subjects. Tibialis muscles were obtained from mice that had been sacrificed for an experiment unrelated to this study. Animal care and use for this experiment were approved by the Animal Welfare Committee of Maastricht University and followed the laws, rules, and guidelines of the Netherlands.

## Author contributions

This study was originally conceived, then initiated and directed by KCO. RJN provided all characterized subject specimens and directed the clinical aspects of the study. KT produced the tetramer. KT and PS performed the single-cell isolation, mAb expression, mAb sequencing, and cell-based antibody assays and interpreted those data. MLF designed and performed additional cell-based antibody assay experiments and contributed to the execution and analysis of the C2C12 assays that PS established and performed. KT, with assistance from MLF and ESB, built and tested the MuSK domain deletion constructs. MC, DB, and AV designed, performed, and interpreted the phosphorylation assays. MMD, PMM, and ML designed and performed the murine muscle staining and interpreted those data. PW, SRI, and LJ initially confirmed mAb specificity, in a blinded manner, by providing independent data from multiple assays. AV and LJ performed and analyzed the radioimmunoassay titration experiments. The manuscript was initially drafted by KCO and AV with substantial input from PS and ML. Key contributions in editing and revising were further provided by PS and ML.

## Supplementary Material

Supplemental data

## Figures and Tables

**Table 2:**
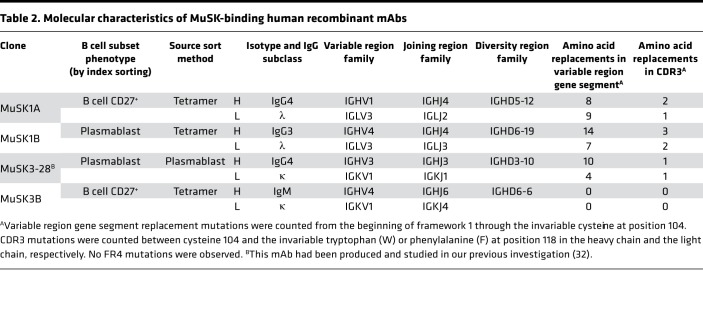
Molecular characteristics of MuSK-binding human recombinant mAbs

**Table 1:**
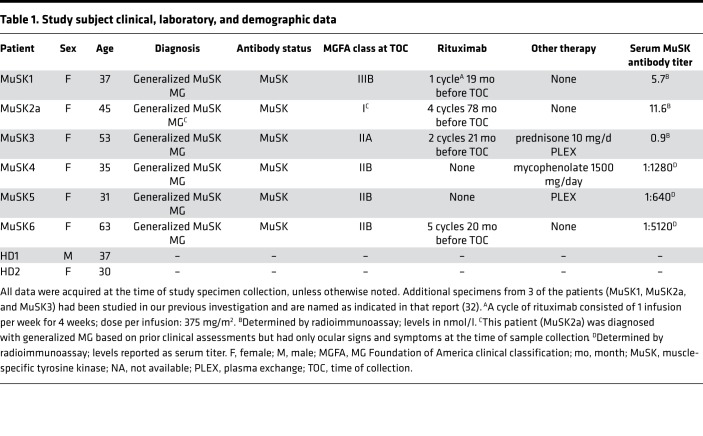
Study subject clinical, laboratory, and demographic data

## Version 1 06/20/2019

Electronic publication
