# Supplementary material for: Characterization of pathogenic monoclonal autoantibodies derived from muscle-specific kinase myasthenia gravis patients
Source: JCI Insight. 2019 Jun 20;4(12):e127167. doi: 10.1172/jci.insight.127167 (PMC6629167; doi:10.1172/jci.insight.127167)
Supplement: Supplemental data [file jciinsight-4-127167-s195.pdf]

Supplemental data:

Characterization of pathogenic monoclonal autoantibodies derived from muscle-specific kinase myasthenia gravis patients

Contents:

Figure S1. Multimerized, labeled MuSK retains properties required for antibody recognition and detection by flow cytometry.

Figure S2. MuSK multimer FACS gating strategy.

Figure S3. MuSK domain expression construct sequences.

Figure S4. Antibody-induced AChR clustering in C2C12 myotubes.

Supplemental methods. B cell isolation, recombinant IgG cloning and expression.

Table S1. Molecular characteristics of human recombinant monoclonal autoantibodies.

Figure S1. Multimerized, labeled MuSK retains properties required for antibody recognition and detection by flow cytometry. Schematic diagram **(A)** showing MuSK expression construct and post-translational tetramer assembly. FACS plots **(B)** showing feasibility of MuSK tetramer-mediated isolation. Flow cytometry beads coated with murine monoclonal antibodies that recognize MuSK (4A3) or myelin oligodendrocyte glycoprotein MOG (8-18C5) were stained with APC-conjugated MuSK multimer. Detection of binding to the beads was analyzed by flow cytometry. Data shown is one of two representative experiments.

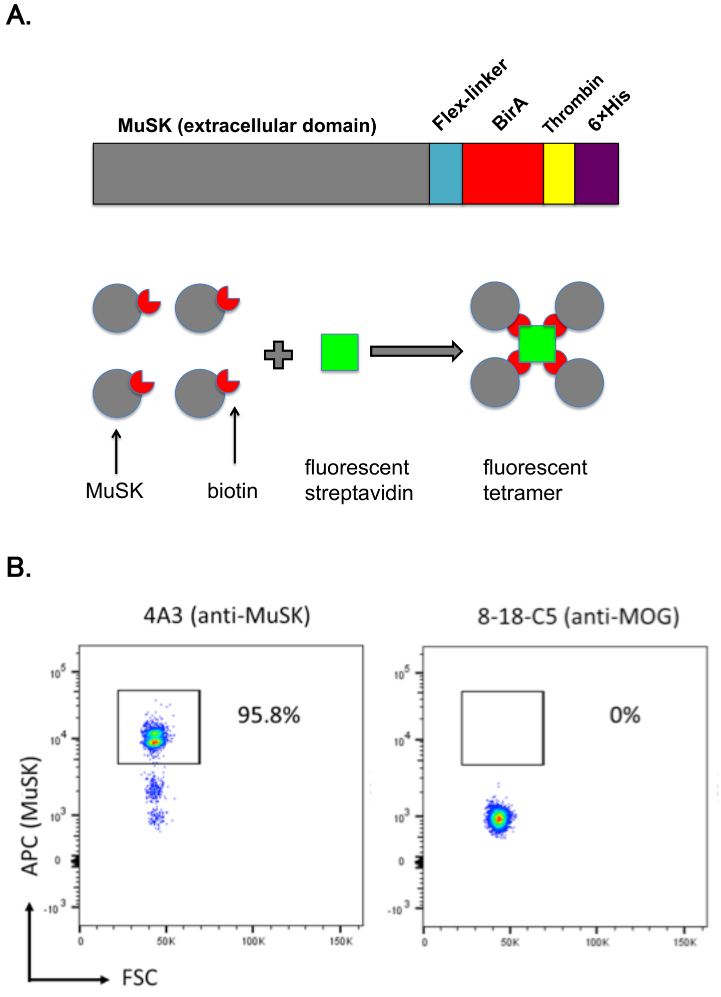

Figure S2. MuSK multimer FACS gating strategy. A representative example of the MuSK multimer positive population sorting strategy is shown. B cells that were enriched through bead-based negative selection, were initially gated in the SSC/FSC graph. After doublets and dead cells were excluded, CD19+CD3-CD14- cells were gated. MuSK multimer positive cells were subsequently gated from the B cell gate (CD19+CD3-CD14-) using CD27+ MuSK multimer+ cells. Plasmablasts were defined as CD27hiCD38hi cells for purposes of back-gating and index-sorting (not shown).

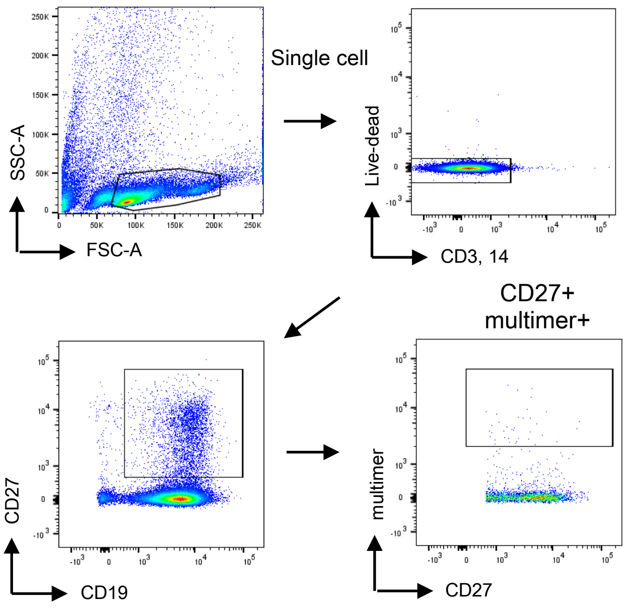

Figure S3. Protein sequences (amino acid) of different MuSK domain expression constructs. Bold letters indicate particular domains. Dots represent deleted regions.

Full length MuSK sequence:

|------------------------------- Ig-like domain-1 -------------------------------|
MRELVNIPLV HILTLVAFSG TEKLPKAPVI TTPLETVDA**L VEEVATFMCA VESYPQPEIS WTRNKILIKL FDTRYSIREN GQLLTILSVE DSDDGIYCCT ANNGVGGAVE SCG**ALQVKMK

|------------------------- Ig-like domain-2 -----------------------------------| |-------------------------------
PKITRPPINV KI**IEGLKAVL PCTTMGNPKP SVSWIKGDSP LRENSRIAVL ESGSLRIHNV QKEDAGQYRC VAKNSLGTAY SKVV**KLEVEV F**ARILRAPES HNVTFGSFVT LHCTATGIPV**

Ig-like domain-3 ---| |------------------------------- Frizzled domain ----
PTITWIENGN AVSSGSIQES VKDRVIDSRL QLFITKPGLY TCIATNKHGE KFSTAKAAAT ISIAEWSKPQ KD**NKGYCAQY RGEVCNAVLA KDALVFLNTS YADPEEAQEL LVHTAWNELK**

--- |
VVSPVCRPAA EALLCNHIFQ ECSPGVVPTP IPICREYCLA VKELFCAKEW LVMEEKTHRG LYRSEMHLLS VPECSKLPSM HWDPTACARL PHLAFPPMTS SKPSVDIPNL PSSSSSSFSV

Ig-like domain 1 deletion:

|------------------------------- Ig-like domain-1 -------------------------------|
MRELVNIPLV HILTLVAFSG TEKLPKAPVI TTPLETVDA. ....ALQVKMK

|------------------------- Ig-like domain-2 -----------------------------------| |-------------------------------
PKITRPPINV KI**IEGLKAVL PCTTMGNPKP SVSWIKGDSP LRENSRIAVL ESGSLRIHNV QKEDAGQYRC VAKNSLGTAY SKVV**KLEVEV F**ARILRAPES HNVTFGSFVT LHCTATGIPV**

Ig-like domain-3 ---| |------------------------------- Frizzled domain ----
PTITWIENGN AVSSGSIQES VKDRVIDSRL QLFITKPGLY TCIATNKHGE KFSTAKAAAT ISIAEWSKPQ KD**NKGYCAQY RGEVCNAVLA KDALVFLNTS YADPEEAQEL LVHTAWNELK**

--- |
VVSPVCRPAA EALLCNHIFQ ECSPGVVPTP IPICREYCLA VKELFCAKEW LVMEEKTHRG LYRSEMHLLS VPECSKLPSM HWDPTACARL PHLAFPPMTS SKPSVDIPNL PSSSSSSFSV

Ig-like domain 2 deletion:

|------------------------------- Ig-like domain-1 -------------------------------|
MRELVNIPLV HILTLVAFSG TEKLPKAPVI TTPLETVDA**L VEEVATFMCA VESYPQPEIS WTRNKILIKL FDTRYSIREN GQLLTILSVE DSDDGIYCCT ANNGVGGAVE SCG**ALQVKMK

|------------------------- Ig-like domain-2 -----------------------------------| |-------------------------------
PKITRPPINV KI........ ....KLEVEV F**ARILRAPES HNVTFGSFVT LHCTATGIPV**

Ig-like domain-3 ---| |------------------------------- Frizzled domain ----
PTITWIENGN AVSSGSIQES VKDRVIDSRL QLFITKPGLY TCIATNKHGE KFSTAKAAAT ISIAEWSKPQ KD**NKGYCAQY RGEVCNAVLA KDALVFLNTS YADPEEAQEL LVHTAWNELK**

--- |
VVSPVCRPAA EALLCNHIFQ ECSPGVVPTP IPICREYCLA VKELFCAKEW LVMEEKTHRG LYRSEMHLLS VPECSKLPSM HWDPTACARL PHLAFPPMTS SKPSVDIPNL PSSSSSSFSV

Frizzled-like domain deletion:

```
                                        |------------------------------- Ig-like domain-1 -------------------------------|
MRELVNIPLV HILTLVAFSG TEKLPKAPVI TTPLETVDAL VEEVATFMCA VESYPQPEIS WTRNKILIKL FDTRYSIREN GQLLTILSVE DSDDGIYCCT ANNGVGGAVE SCGALQVKMK

            |------------------------- Ig-like domain-2 ----------------------------------|      |------------------------------
PKITRPPINV KIIEGLKAVL PCTTMGNPKP SVSWIKGDSP LRENSRIAVL ESGSLRIHNV QKEDAGQYRC VAKNSLGTAY SKVVKLEVEV FARILRAPES HNVTFGSFVT LHCTATGIPV

Ig-like domain-3 ---------------------------------------------------|         |------------------------------ Frizzled domain ----
PTITWIENGN AVSSGSIQES VKDRVIDSRL QLFITKPGLY TCIATNKHGE KFSTAKAAAT ISIAEWSKPQ KD........ .......... .......... .......... ..........

--------------------------------------------------------------------------------------------------- |
.......... .......... .......... .......... .......... .......... .......... .......... .......... PHLAFPPMTS SKPSVDIPNL PSSSSSSFSV
```

Ig-like domain 1 only:

```
                                        |------------------------------- Ig-like domain-1 -------------------------------|
MRELVNIPLV HILTLVAFSG TEKLPKAPVI TTPLETVDAL VEEVATFMCA VESYPQPEIS WTRNKILIKL FDTRYSIREN GQLLTILSVE DSDDGIYCCT ANNGVGGAVE SCGALQVKMK

            |------------------------- Ig-like domain-2 ----------------------------------|      |------------------------------
.......... .......... .......... .......... .......... .......... .......... .......... .......... .......... .......... ..........

Ig-like domain-3 ---------------------------------------------------|         |------------------------------ Frizzled domain ----
.......... .......... .......... .......... .......... .......... .......... .......... .......... .......... .......... ..........

--------------------------------------------------------------------------------------------------- |
.......... .......... .......... .......... .......... .......... .......... .......... .......... PHLAFPPMTS SKPSVDIPNL PSSSSSSFSV
```

Ig-like domain 2 only:

```
                                        |------------------------------- Ig-like domain-1 -------------------------------|
MRELVNIPLV HILTLVAFSG TEKLPKAPVI TTPLETVDA. .......... .......... .......... .......... .......... .......... .......... ...ALQVKMK

            |------------------------- Ig-like domain-2 ----------------------------------|      |------------------------------
PKITRPPINV KIIEGLKAVL PCTTMGNPKP SVSWIKGDSP LRENSRIAVL ESGSLRIHNV QKEDAGQYRC VAKNSLGTAY SKVVKLEVEV F......... .......... ..........

Ig-like domain-3 ---------------------------------------------------|         |------------------------------ Frizzled domain ----
.......... .......... .......... .......... .......... .......... .......... .......... .......... .......... .......... ..........

--------------------------------------------------------------------------------------------------- |
.......... .......... .......... .......... .......... .......... .......... .......... .......... PHLAFPPMTS SKPSVDIPNL PSSSSSSFSV
```

Frizzled-like domain only:

|------------------------------- Ig-like domain-1 -------------------------------|
MRELVNIPLV HILTLVAFSG TEK....... ....

|------------------------- Ig-like domain-2 ----------------------------------| |------------------------------
....

Ig-like domain-3 --| |------------------------------ Frizzled domain ----
....SKPQ KD**NKGYCAQY RGEVCNAVLA KDALVFLNTS YADPEEAQEL LVHTAWNELK**

-- |
VVSPVCRPAA EALLCNHIFQ ECSPGVVPTP IPICREYCLA VKELFCAKEW LVMEEKTHRG LYRSEMHLLS VPECSKLPSM HWDPTACARL PHLAFPPMTS SKPSVDIPNL PSSSSSSFSV

Figure S4. Antibody-induced AChR clustering in C2C12 mouse myotubes. The presence of agrin in C2C12 myotube cultures leads to dense clustering of AChRs that can be readily visualized with fluorescent α-bungarotoxin and quantified. Pathogenic MuSK autoantibodies can disrupt this clustering (*see main text and figures*). Three different human MuSK-specific mAbs (MuSK1A, MuSK1B, and MuSK3-28), the humanized murine control MuSK mAb 4A3 and three human non- MuSK-specific mAbs (64-2, 64-7, 64-8) were tested for their ability to induce the AChR clustering in the absence of agrin. Clustering of AChR was quantified relative to the measured effect of agrin. Quantitative results are normalized to agrin-only induced clustering. Each mAb was added to the cultures at 1μg/mL. Each data point represents the mean value from an independent experiment. Bars represent the mean of means and error bars SDs. Multiple comparisons ANOVA (against agrin), Dunnet's test; * p<0.05, ** p<0.01, *** p<0.001, **** p<0.0001, only shown when significant).

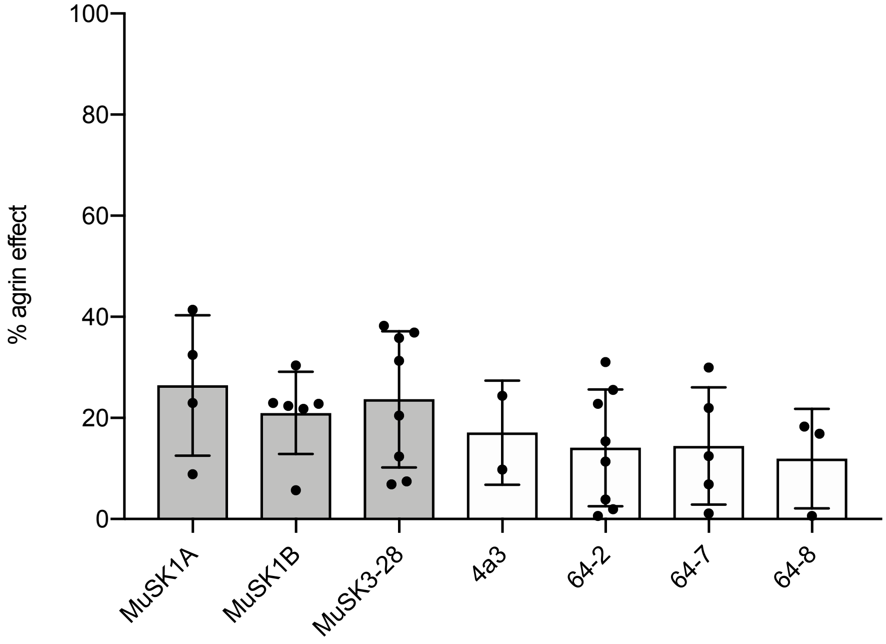

Supplemental methods. Recombinant IgG cloning and expression.

Single cell RT-PCR and Ig gene amplification

Single human B cells were isolated as described in the Methods section of the main text. Variable region domains from all cells were amplified using an established RT-PCR approach (Tiller *et al.*, 2008). Total RNA from single cells was reverse transcribed in nuclease-free water using 150 ng random hexamer primer (pd(N)6, GE Healthcare), 0.5 µl of 10 mM each nucleotide dNTP-Mix (Invitrogen), 1 µl 0.1 M DTT (Invitrogen), 0.5% v/v Igepal CA-630 (Sigma), 4 U RNAsin® (Promega), 6 U Prime RNAse Inhibitor™ (Eppendorf) and 50 U Superscript® III reverse transcriptase (Invitrogen). Reverse transcription (RT) reaction was performed at 42 °C for 10 min, 25 °C for 10 min, 50 °C for 60 min and 94 °C for 5 min. Synthesis of cDNA was carried out in a total volume of 14 µl/well in the original 96-well sorting plate. cDNA was stored at − 20 °C. IgH, Igλ and Igκ V gene transcripts were amplified independently by a nested PCR starting from 3.5 µl of cDNA as template. All PCR reactions were performed in 96-well plates in a total volume of 42 µl per well containing 20 nM each primer or primer mix (oligonucleotide primer sequences as previously described (Tiller *et al.*, 2008), 10 uM each dNTP (Invitrogen) and 1.2 U HotStar® Taq DNA polymerase (Qiagen). All nested PCR reactions with gene-specific primers or primer mixes were performed with 4 µl of unpurified first PCR product. Each round of PCR was performed for 50 cycles at 94 °C for 30 s, 59 °C (IgH/Igκ) or 60 °C (Igλ) for 30 s, 72 °C for 55 s (1st PCR) or 45 s (2nd PCR).

Immunoglobulin gene sequence analysis

Aliquots of the VH, Vκ and Vλ chain nested PCR products were purified with QIAquick PCR Purification Kit (Qiagen) according to the manufacturer's instructions. These products were then sequenced with the respective reverse primer (Tiller *et al.*, 2008). Sequences were analyzed by IMGT/HighV-QUEST. The heavy- and light-chain variable region germline gene segments were assigned with IMGT/HighV-QUEST (http://imgt.org) (Alamyar *et al.*, 2012) using the July 7, 2015, version of the IMGT gene database. Somatic mutations resulting in replacement amino acids were evaluated through the alignment to germline genes provided by the IMGT V-base algorithm. The PCR sequencing step was verified by sequencing of the plasmids containing the sub-cloned variable heavy and light chain domains (Tiller *et al.*, 2008).

Immunoglobulin gene IgG subclass by constant region sequencing.

To determine the immunoglobulin IgG subclass, two strategies were used. First, the product of the first heavy chain PCR was used in a second PCR with the IgG internal reverse primer (5'-GTTCGGGGAAGTAGTCCTTGAC-3' (Tiller *et al.*, 2008). Second, a nested PCR was performed as described above starting with cDNA and using the same forward primers (Tiller *et al.*, 2008) and a different reverse primer (5'-GGGGAAGTAGTCCTTGACCAG-3', (Maillette de Buy Wenniger *et al.*, 2013). The PCR product was purified and send to sequencing.

Expression vector cloning IgG1, kappa and lambda.

All single-cell PCR products were purified using the QIAquick PCR Purification Kit (Qiagen) prior to subcloning. Volumes obtained after elution were 30 µl. Digestions were carried out with the respective restriction enzymes AgeI, BsiWI, SalI and XhoI (all from NEB) in a total volume of 35–40 µl (Tiller *et al.*, 2008). Digested PCR products were purified as described above (Qiagen) before ligation into human

Igγ1, Igκ and Igλ expression vectors containing a murine Ig gene signal peptide sequence (GenBank accession no. DQ407610) and a multiple cloning site upstream of the human Igγ1 (IGHG1), Igκ (IGKC) or Igλ (IGLC2) constant regions. Transcription is under the influence of the human cytomegalovirus (hCMV) promotor and clones can be selected based on resistance to ampicillin. Ligation was performed in a total volume of 10 µl with 1 U T4 DNA-Ligase (Invitrogen), 7.5 µl of digested and purified PCR product and 25 ng linearized vector. Competent E. coli DH10B bacteria (Clontech) were transformed at 42 °C with 4.5 µl of the ligation product. Colonies were screened by PCR using 5′Absense as forward primer and 3′IgGinternal, 3′Cκ494 or 3′Cλ as reverse primer, respectively (Tiller *et al.*, 2008). PCR products of the expected size (650 bp for Igγ1, 700 bp for Igκ and 590 bp for Igλ) were sequenced to confirm identity with the original PCR products. Plasmid DNA was isolated from 4 ml bacteria cultures grown for 16 h at 37 °C in Terrific Broth (Difco Laboratories) containing 75 µg/ml ampicillin (Sigma) using QIAprep Spin columns (Qiagen). All plasmids were sequenced to validate that the correct variable regions were subcloned and that open reading frames (from the signal peptide through to the Ig constant domain) were present.

Expression vector cloning IgG4.

For initial antigen specificity screening, all of the variable heavy chain domains were cloned into the human IgG1 subclass expression vector (described above), irrespective of their native isotype or IgG subclass usage. Variable heavy chain domains that were derived from cells expressing IgG4 were cloned, in addition to the IgG1 vector, into a human IgG4 subclass expression vector. PCR-amplified variable heavy chain domains were directionally sub-cloned downstream of the CMV promoter into a pcDNA3.3-based vector (selected based on resistance to kanamycin or ampicillin) generated in our laboratory to harbor the human immunoglobulin constant domain for IgG4. The variable heavy chain domains were cloned into a cassette downstream of a signal peptide for prolactin (UniProtKB - P01239 (PRL_BOVIN)), MDSKGSSQKGSRLLLLLVVSNLLLCQGVVS, followed by an Afe I site (at the 5’ end of the VH domain) and Apa I present at the 5’ end of the IgG4 constant domain. Digests were carried out with the respective restriction enzymes and digested PCR products were sub-cloned and validated as described above.

Recombinant human monoclonal antibody production

Human embryonic kidney (HEK) 293A (ThermoFisher R70507) cells were cultured in 100 mm plates (Falcon, Becton Dickinson) under standard conditions in Dulbecco's Modified Eagle's Medium (DMEM; 12030 Gibco) supplemented with 10% certified fetal calf serum (FCS) (Gibco 16000), 1% non-essential amino acids (Gibco), 1% P/S (Penicillin-Streptomycin (10,000 U/mL) (Gibco), 2mM L-Glutamine (Gibco).

Transient transfections of exponentially growing 293A cells were performed by using linear polyethylenimine (Polysciences Cat# 23966) for 16 h at approximately 80-90% cell confluency. Equal amounts (3µg each) of IgH and corresponding IgL chain expression vector DNA were added to 800µL of DMEM containing PEI. This solution was vortexed, then allowed to incubate at room temperature for 15 minutes, before being evenly distributed in the culture dish. 24 h later the tranfection media was removed and the cells were cultured for 6 days in 8 ml Basal media (DMEM (Gibco, 12430) 50%, RPMI (Gibco, 11875) 50%, A/A 0.75% (ThermoFisher, 15240062), L-glutamine 0.2mM (Gibco, 25030081), Sodium-Pyruvat 1% (ThermoFisher, 11360070) supplemented with 1% Nutridoma-SP (Roche,

11363743001). Cells and cell debris was then removed by centrifugation at 800 ×g for 10 min and culture supernatants were stored at 4°C with prior to Ig purification (below).

Recombinant human monoclonal antibody purification

Recombinant antibodies were purified with Protein G Sepharose 4 Fast Flow beads (GE Healthcare, 17061801). In brief, approximately 25 ml cell culture supernatants were incubated with 200 µl Protein G beads for at least 48 h overnight at 4 °C under rotation. Supernatants were removed after centrifugation at 1500rpm for 10 min and the beads were transferred to a chromatography spin column (BioRad) equilibrated with PBS. After two rounds of washing with 1 ml PBS, antibodies were eluted in 3–4 fractions (200 µl each) with 0.1 M glycine (pH 3.0). Eluates were collected in tubes containing 20 µl 1 M Tris (pH 8.0) and 20 ul of 10xPBS (pH 7.4). The pH was confirmed to be near neutral (pH 7.4) using narrow range (pH 5.0 - 10) indicator strips. Purified recombinant human monoclonal antibody concentrations were determined by measuring absorption at 280 nm with a microvolume UV-Vis spectrophotometer (NanoDrop 2000, ThermoFisher). As an additional means to validate expression, only purified recombinant immunoglobulins that were both above 50µg/mL and generated an expected UV spectral curve shape (250-300nm) for purified proteins were used for subsequent experiments.

Table S1. Molecular characteristics of human recombinant monoclonal autoantibodies.

Variable region gene segment replacement mutations were counted from the beginning of frame-work 1 through the invariable cysteine at position 104. CDR3 mutations were counted between cysteine 104 and the invariable tryptophan (W) or phenylalanine (F) at position 118 in the heavy chain and the light chain respectively. FR4 mutations were not considered. ND, not determined.

Clone		Isotype and IgG subclass	Variable region family	Joining region family	Diversity region family	Amino acid replacements in variable region gene segment[a]	Amino acid replacements in CDR3[a]
MuSK1A	H	IgG4	IGHV1	IGHJ4	IGHD5	8	2
	L	λ	IGLV3	IGLJ2		1	0
MuSK1B	H	IgG3	IGHV4	IGHJ4	IGHD6	15	3
	L	λ	IGLV3	IGLJ3		8	2
MuSK3-28	H	IgG4	IGHV3	IGHJ3	IGHD3	10	0
	L	κ	IGKV1	IGKJ1		4	1
MuSK3B	H	M	IGHV4	IGHJ6	IGHD6	0	0
	L	κ	IGKV1	IGKJ4		0	0
MuSK1-2	H	M	IGHV3	IGHJ6	IGHD3	0	0
	L	λ	IGLV3	IGLJ2		1	0
MuSK1-3	H	M	IGHV3	IGHJ6	IGHD2	1	2
	L	κ	IGKV4	IGKJ3		1	0
MuSK1-4	H	M	IGHV3	IGHJ4	IGHD5	2	0
	L	λ	IGLV4	IGLJ3		0	0
MuSK1-6	H	M	IGHV3	IGHJ3	IGHD3	2	0
	L	λ	IGLV2	IGLJ1		0	0
MuSK1-7	H	ND	IGHV3	IGHJ6	IGHD2	0	0
	L	κ	IGKV1	IGKJ2		0	0
MuSK1-8	H	A	IGHV1	IGHJ4	IGHD1	0	0
	L	λ	IGLV2	IGLJ3		0	0
MuSK1-9	H	M	IGHV3	IGHJ3	IGHD3	1	1
	L	κ	IGKV1	IGKJ1		0	0
MuSK1-12	H	M	IGHV3	IGHJ4	IGHD3	2	1
	L	κ	IGKV2	IGKJ1		ND	1
MuSK1-17	H	M	IGHV4	IGHJ4	IGHD5	0	0
	L	λ	IGLV9	IGLJ3		0	0
MuSK1-18	H	M	IGHV1	IGHJ6	IGHD3	0	0
	L	κ	IGKV2	IGKJ5		0	0
MuSK1-20	H	ND	IGHV1	IGHJ6	IGHD3	0	0

	L	λ	IGLV2	IGLJ2 or IGLJ3		0	0
MuSK1-22	H	ND	IGHV1	IGHJ6	IGHD4	0	0
	L	λ	IGLV3	IGLJ2 or IGLJ3		0	0
MuSK1-29	H	M	IGHV4	IGHJ4	IGHD6	0	0
	L	κ	IGKV1	IGKJ4		0	0
MuSK1-30	H	M	IGHV4	IGHJ6	IGHD3	0	1
	L	λ	IGLV3	IGLJ2 or IGLJ3		0	0
MuSK1-33	H	ND	IGHV5	IGHJ4	IGHD6	1	0
	L	κ	IGKV2	IGKJ1		0	0
MuSK1-36	H	ND	IGHV3	IGHJ6	IGHD3	0	0
	L	λ	IGLV1	IGLJ1		0	0
MuSK1-41	H	IgG1	IGHV3	IGHJ5	IGHD2	0	1
	L	κ	IGKV3	IGKJ4		2	0
MuSK1-42	H	M	IGHV3	IGHJ6	IGHD2	0	0
	L	κ	IGKV1	IGKJ1		0	0
MuSK1-44	H	M	IGHV3	IGHJ4	IGHD3	0	0
	L	κ	IGKV1	IGKJ4		0	0
MuSK1-46	H	ND	IGHV4	IGHJ4	IGHD3	0	1
	L	κ	IGKV3	IGKJ4		0	0
MuSK2a-2	H	ND	IGHV4	IGHJ4	IGHD1	0	0
	L	κ	IGKV3	IGKJ1		0	0
MuSK2a-7	H	ND	IGHV4	IGHJ4	IGHD4	1	0
	L	ND	IGLV7	ND		5	ND
MuSK2a-10	H	ND	IGHV4	IGHJ5	IGHD3	9	2
	L	κ	IGKV4	IGKJ2		4	4
MuSK2a-16	H	ND	IGHV3	IGHJ4	IGHD5	3	0
	L	λ	IGLV2	IGLJ3		0	1
MuSK2a-17	H	ND	IGHV3	IGHJ4	IGHD5	10	5
	L	λ	IGLV2	IGLJ3		5	0
MuSK5-1	H	M	IGHV3	IGHJ4	IGHD4	5	2
	L	κ	IGKV3	IGLJ2		9	0
MuSK5-2	H	ND	IGHV4	IGHJ2	IGHD4	2	2
	L	κ	IGKV1	IGKJ1		4	0
MuSK5-5	H	IgG2	IGHV3	IGHJ4	IGHD6	6	2
	L	λ	IGLV2	IGLJ3		0	3
MuSK5-6	H	ND	IGHV1	IGHJ5	IGHD4	12	2

	L	κ	IGKV1	IGKJ3		6	1
MuSK5-7	H	ND	IGHV3	IGHJ6	IGHD2	4	3
	L	κ	IGKV1	IGKJ3		3	1
MuSK5-8	H	ND	IGHV1	IGHJ5	IGHD3	0	2
	L	λ	IGLV1	IGLJ3		0	0
MuSK5-9	H	ND	IGHV4	IGHJ5	IGHD3	8	1
	L	κ	IGKV1	IGKJ2		9	2
MuSK5-11	H	ND	IGHV3	IGHJ4	IGHD3	9	3
	L	κ	IGKV1	IGKJ5		4	2
MuSK5-13	H	ND	IGHV4	IGHJ6	IGHD6	2	2
	L	κ	IGKV1	IGKJ2		2	0
MuSK5-15	H	ND	IGHV4	IGHJ4	IGHD3	7	3
	L	κ	IGKV2	IGKJ2		3	0
MuSK5-17	H	ND	IGHV3	IGHJ6	IGHD3	12	1
	L	κ	IGKV3	IGKJ1		6	2
MuSK5-18	H	IgG1	IGHV4	IGHJ6	IGHD3	11	2
	L	κ	IGKV3	IGKJ2		7	2
MuSK5-21	H	ND	IGHV4	IGHJ6	IGHD2	5	3
	L	κ	IGKV2	IGKJ5		3	1
MuSK5-22	H	ND	IGHV4	IGHJ4	IGHD3	2	2
	L	κ	IGKV1	IGKJ1		0	0
MuSK5-23	H	ND	IGHV4	IGHJ6	IGHD5	2	1
	L	κ	IGKV1	IGKJ1		0	0
MuSK5-26	H	ND	IGHV3	IGHJ4	IGHD2	6	0
	L	κ	IGKV1	IGKJ2		8	1
MuSK5-28	H	ND	IGHV3	IGHJ2	IGHD1	9	3
	L	κ	IGKV1	IGKJ2		8	0
MuSK5-30	H	ND	IGHV3	IGHJ4	IGHD2	5	3
	L	κ	IGKV1	IGKJ4		7	1
MuSK5-31	H	ND	IGHV4	IGHJ4	IGHD3	7	4
	L	κ	IGKV4	IGKJ1		3	0
MuSK5-34	H	ND	IGHV3	IGHJ4	IGHD3	7	2
	L	κ	IGKV2	IGKJ2		5	1
MuSK5-35	H	ND	IGHV3	IGHJ2	IGHD6	19	2
	L	κ	IGKV2	IGKJ1		5	0
MuSK4-1	H	ND	IGHV3	IGHJ2	IGHD2	0	0
	L	κ	IGKV3	IGKJ1		0	0
MuSK4-3	H	ND	IGHV3	IGHJ4	IGHD1	0	0
	L	κ	IGKV3	IGKJ1		0	0

MuSK4-4	H	ND	IGHV4	IGHJ6	IGHD6	0	0
	L	λ	IGLV1	IGLJ3		0	0
MuSK4-8	H	ND	IGHV3	IGHJ3	IGHD6	5	1
	L	κ	IGKV3	IGKJ2		2	0
MuSK4-11	H	ND	IGHV4	IGHJ5	IGHD3	3	2
	L	κ	IGKV4	IGKJ1		2	2
MuSK4-12	H	ND	IGHV1	IGHJ4	IGHD3	1	1
	L	λ	IGLV2	IGLJ2 or IGLJ3		0	0
MuSK4-14	H	ND	IGHV3	IGHJ4	IGHD3	0	0
	L	κ	IGKV1	IGKJ1		0	0
MuSK4-18	H	ND	IGHV3	IGHJ4	IGHD6	9	1
	L	κ	IGKV1	IGKJ1		2	0
MuSK4-19	H	ND	IGHV4	IGHJ4	IGHD3	11	0
	L	λ	IGLV2	IGLJ2 or IGLJ3		4	2
MuSK4-22	H	ND	IGHV3	IGHJ3	IGHD6	1	1
	L	λ	IGLV1	IGLJ2 or IGLJ3		1	2
MuSK4-23	H	ND	IGHV1	IGHJ5	IGHD5	0	0
	L	λ	IGLV2	IGKJ1		0	0
MuSK4-26	H	ND	IGHV1	IGHJ6	IGHD2	0	0
	L	λ	IGLV2	IGLJ2 or IGLJ3		0	0
MuSK4-31	H	M	IGHV3	IGHJ3	IGHD5	0	1
	L	κ	IGKV1	IGKJ1		2	0
MuSK3-1	H	ND	IGHV4	IGHJ5	IGHD5	1	0
	L	κ	IGKV3	IGKJ1		0	0
MuSK3-6	H	ND	IGHV1	IGHJ1	IGHD6	1	0
	L	κ	IGKV1	IGKJ1		0	0
MuSK3-13	H	IgG1	IGHV3	IGHJ3	IGHD5	5	1
	L	λ	IGLV3	IGLJ2 or IGLJ3		2	1
MuSK3-16	H	IgG1	IGHV1	IGHJ1	IGHD2	15	3
	L	κ	IGKV1	IGKJ2		13	2
MuSK3-19	H	ND	IGHV3	IGHJ6	IGHD6	1	0
	L	κ	IGKV2	IGKJ4		0	0
MuSK3-20	H	ND	IGHV3	IGHJ4	IGHD2	0	1
	L	κ	IGKV1	IGKJ1		1	0

MuSK3-22	H	ND	IGHV4	IGHJ4	IGHD1	6	0
	L	λ	IGLV1	IGLJ2 or IGLJ3		3	0
MuSK3-23	H	ND	IGHV4	IGHJ2	IGHD3	0	0
	L	κ	IGKV1	IGKJ2		0	0
MuSK3-24	H	ND	IGHV3	IGHJ4	IGHD3	0	1
	L	κ	IGKV3	IGKJ1		0	0
MuSK3-25	H	ND	IGHV4	IGHJ5	IGHD3	0	0
	L	κ	IGKV1	IGKJ1		1	0
MuSK6-2	H	M	IGHV1	IGHJ4	IGHD6	0	1
	L	κ	IGKV1	IGKJ3		0	0
MuSK6-5	H	M	IGHV4	IGHJ4	IGHD3	0	0
	L	λ	IGLV2	IGLJ2 or IGLJ3		1	0
MuSK6-12	H	M	IGHV3	IGHJ4	IGHD2	2	0
	L	λ	IGLV2	IGLJ3		1	0
MuSK6-13	H	IgG1	IGHV4	IGHJ6	IGHD1	8	1
	L	λ	IGLV2	IGLJ2 or IGLJ3		3	1
HD1-4	H	M	IGHV3	IGHJ4	IGHD1	7	2
	L	λ	IGLV1	IGLJ2 or IGLJ3		3	2
HD1-6	H	M	IGHV3	IGHJ4	IGHD3	0	2
	L	κ	IGKV3	IGKJ4		3	0
HD1-9	H	ND	IGHV1	IGHJ6	IGHD5	2	1
	L	λ	IGLV2	IGLJ3		3	1
HD1-10	H	M	IGHV1	IGHJ6	IGHD3	0	0
	L	λ	IGLV2	IGLJ2 or IGLJ3		3	0
HD1-11	H	M	IGHV3	IGHJ4	IGHD2	5	2
	L	κ	IGKV1	IGKJ4		2	0
HD1-20	H	M	IGHV3	IGHJ5	IGHD6	0	0
	L	κ	IGKV1	IGKJ4		0	0
HD1-21	H	M	IGHV3	IGHJ6	IGHD4	4	0
	L	κ	IGKV1	IGKJ1		1	0
HD1-28	H	ND	IGHV3	IGHJ4	IGHD5	1	1
	L	λ	IGLV2	IGLJ1		4	1
HD1-29	H	MND	IGHV3	IGHJ4	IGHD3	5	2
	L	κ	IGKV1	IGKJ3		0	0

HD1-30	H	M	IGHV3	IGHJ3	IGHD1	10	3
	L	κ	IGKV4	IGKJ1		1	0
HD2-3	H	ND	IGHV3	IGHJ4	IGHD3	14	4
	L	κ	IGKV3	IGKJ4		7	1
HD2-4	H	ND	IGHV1	IGHJ5	IGHD3	12	1
	L	κ	IGKV3	IGKJ4		4	0
HD2-6	H	ND	IGHV4	IGHJ3	IGHD1	3	0
	L	κ	IGKV3	IGKJ1		3	0
HD2-7	H	M	IGHV3	IGHJ4	IGHD5	0	0
	L	κ	IGKV3	IGKJ4		0	0
HD2-8	H	M	IGHV3	IGHJ4	IGHD4	7	1
	L	κ	IGKV3	IGKJ4		1	0
HD2-15	H	ND	IGHV3	IGHJ4	IGHD1	7	0
	L	λ	IGLV2	IGLJ2 or IGLJ3		3	0
HD2-16	H	ND	IGHV3	IGHJ4	IGHD4	4	0
	L	κ	IGKV1	IGKJ4		5	0
HD2-18	H	ND	IGHV3	IGHJ4	IGHD5	7	2
	L	κ	IGKV1	IGKJ4		6	1
HD2-19	H	ND	IGHV3	IGHJ4	IGHD1	5	1
	L	κ	IGKV1	IGKJ3		8	2
HD2-20	H	M	IGHV3	IGHJ4	IGHD2	5	2
	L	λ	IGLV2	IGLJ1		3	0
HD2-26	H	ND	IGHV4	IGHJ4	IGHD5	6	1
	L	λ	IGLV2	IGLJ1		3	1
HD2-27	H	ND	IGHV3	IGHJ4	IGHD4	4	0
	L	κ	IGKV3	IGKJ4		1	1
HD2-28	H	M	IGHV3	IGHJ4	IGHD6	6	1
	L	λ	IGLV7	IGLJ3		2	0
HD2-31	H	ND	IGHV3	IGHJ4	IGHD3	0	0
	L	κ	IGKV1	IGKJ2		0	0
HD2-32	H	ND	IGHV3	IGHJ5	IGHD6	5	3
	L	λ	IGLV2	IGLJ3		2	2
HD2-33	H	ND	IGHV3	IGHJ4	IGHD4	3	0
	L	λ	IGLV2	IGLJ3		6	0
HD2-34	H	ND	IGHV3	IGHJ4	IGHD3	1	1
	L	λ	IGLV2	IGLJ2 or IGLJ3		2	0
HD2-35	H	M	IGHV1	IGHJ4	IGHD3	6	1

	L	λ	IGLV2	IGLJ1		4	0
HD2-37	H	M	IGHV4	IGHJ6	IGHD2	1	1
	L	κ	IGKV1	IGKJ1		0	0

[a]Variable region gene segment replacement mutations were counted from the beginning of frame-work 1 through the invariable cysteine at position 104. CDR3 mutations were counted between cysteine 104 and the invariable tryptophan (W) or phenylalanine at position 118 in the heavy chain and the light chain respectively. No FR4 mutations were observed.

References

Alamyar E, Duroux P, Lefranc MP, Giudicelli V. IMGT((R)) tools for the nucleotide analysis of immunoglobulin (IG) and T cell receptor (TR) V-(D)-J repertoires, polymorphisms, and IG mutations: IMGT/V-QUEST and IMGT/HighV-QUEST for NGS. Methods Mol Biol 2012; 882: 569-604.

Maillette de Buy Wenniger LJ, Doorenspleet ME, Klarenbeek PL, Verheij J, Baas F, Elferink RP*, et al.* Immunoglobulin G4+ clones identified by next-generation sequencing dominate the B cell receptor repertoire in immunoglobulin G4 associated cholangitis. Hepatology 2013; 57(6): 2390-8.

Tiller T, Meffre E, Yurasov S, Tsuiji M, Nussenzweig MC, Wardemann H. Efficient generation of monoclonal antibodies from single human B cells by single cell RT-PCR and expression vector cloning. J Immunol Methods 2008; 329(1-2): 112-24.
